# Neuroprotective effects of a novel peptide through the Rho-integrin-Tie2 and PI3K/Akt pathways in experimental autoimmune encephalomyelitis model

**DOI:** 10.3389/fphar.2024.1290128

**Published:** 2024-02-06

**Authors:** Wen Zhou, Han Qu, Xiao-Xiao Fu, Miao-Miao Xu, Qiang Li, Yuan Jiang, Shu Han

**Affiliations:** ^1^ Department of Emergency Medicine, The Second Affiliated Hospital of Zhejiang University School of Medicine, Hangzhou, China; ^2^ Institute of Anatomy and Cell Biology, Medical College, Zhejiang University, Hangzhou, China; ^3^ Institute of Human Anatomy, Histology and Embryology, Basic Medical College, Zhejiang Chinese Medical University, Hangzhou, China; ^4^ Department of Rehabilitation in Traditional Chinese Medicine, The Second Affiliated Hospital of Zhejiang University School of Medicine, Hangzhou, China; ^5^ Department of Pulmonology, Children’s Hospital, Zhejiang University School of Medicine, National Clinical Research Center for Child Health, Hangzhou, China

**Keywords:** C16, EAE, Tie2, integrin, PI3K/Akt pathways

## Abstract

**Purpose:**

The interaction between inflammatory cells and integrin in the endothelium plays a key role during infiltration. Previous evidence has shown that synthetic C16 peptide selectively binds to integrins αvβ3 and α5β1 and exhibits a neuroprotective effect. It has also been reported to inhibit the differentiation of microglia into the M1 (pro-inflammatory) phenotype while promoting its differentiation to the M2 (anti-inflammatory) phenotype. This study aimed to investigate the mechanisms of action of the C16 peptide in multiple sclerosis using a rodent model.

**Methods:**

Molecular, morphological, and neurophysiological assays were used to investigate the neuroprotective effects of C16 peptide and related signaling pathways in a model of EAE.

**Results:**

The results showed that C16 significantly improved the clinical score and cortical somatosensory/motor evoked potential. It also alleviated inflammatory responses, including microglial activation and leukocyte infiltration, relieved the impairment of the brain blood barrier and edema, and reduced neuronal apoptosis, axonal loss, and demyelination induced by EAE. The C16 peptide increased the expressions of pTie-2 and Tie-2, integrin αvβ3, and α5β1 and activated the PI3K/Akt signal pathway but decreased the expression of Rho. Co-treatment of C16 with Tie-2 inhibitor and PI3K inhibitor LY294002 attenuated these effects of C16.

**Conclusion:**

The C16 peptide demonstrated neuroprotection in the EAE model through the integrin, Tie-2, and PI3K/Akt signaling pathways, and it could be a potential strategy for treating inflammation-related diseases in the central nervous system.

## 1 Introduction

Multiple sclerosis (MS) is an immune-mediated neurodegenerative disease of the central nervous system (CNS) and has some unique features, including infiltration of inflammatory cells, accumulation of demyelinating plaques, and damage to the axon (Constantinescu et al., 2011; Kempuraj et al., 2016). Experimental autoimmune encephalomyelitis (EAE) is a useful MS model (Procaccini et al., 2015). An EAE model of Lewis rats induced by guinea pig spinal cord homogenate (GPSCH) is characterized by a reversible disability (self-recovery trend). The animal clinical score will increase to get an acute phase peak at 2 weeks post-immunization and then gradually recover spontaneously, allowing us to elucidate the induction, peak, and resolution of the inflammation-based immune response of the MS. Currently, the intervention of neuroinflammation and neurodegeneration in the damaged regions of CNS is the major therapeutic strategy for MS (Arnon and Aharoni, 2009). To reach the inflammatory regions of the CNS, leukocytes need to tether and roll along the blood vessels, adhere to and migrate out of the blood vessels. The interaction between leukocytes and the endothelium during this infiltration process plays a key role (Weerasinghe et al., 1998). At the same time, activated microglia could also produce a pro-inflammatory milieu, strip off myelin from neuronal axons, and attract activated T-lymphocytes, further augmenting myelin’s destruction. Therefore, protecting the integrity of the endothelium in the CNS and suppressing the activation of microglia may be targets for therapeutic strategies of MS.

The synthetic C16 peptide (KAFDITYVRLKF) can selectively bind to ανβ3 and α5β1 integrins as a functioning laminin domain (Han et al., 2010). These integrins can regulate the adhesion of leukocytes to the intercellular adhesion molecule-1 (ICAM-1) and thus promote the migration of leukocytes across the endothelium (Han et al., 2010). Similar to the effects of ανβ3 integrin antibody shown in an *in vitro* study, C16 can decrease the transmigration of monocytes to pass across the endothelium layer (Han et al., 2010). However, our *in vitro* data suggested that C16 peptide not only occupies the integrin-binding sites on inflammatory cells and/or vascular endothelium but also reduces the polarization of microglia to the M1 pro-inflammatory phenotype and induces its polarization to the M2 anti-inflammatory phenotype (Fu et al., 2020). We hypothesized that the signaling pathways underlying this phenomenon may be mediated by Tie2-PI3K/Akt, Tie2-integrin, and integrin-PI3K/Akt.

## 2 Materials and methods

### 2.1 Establishment of the EAE model and animals treatment

Adult male Lewis rats (250 g, 10 weeks old, n = 90) were purchased from the Services Centre of Laboratory Animals of Zhejiang University and randomly assigned to the following groups: normal control group (n = 10), vehicle group (n = 20), C16 group (n = 20), C16 + Tie-2 inhibitor group (n = 20), and C16 + LY294002 group (n = 20). The EAE model was established by subcutaneous injection of 0.2 mL of a mixture of guinea pig spinal cord homogenate (GPSCH, 0.1 mL), complete Freud adjuvant (CFA, 0.1 mL, Sigma-Aldrich, St. Louis, MO, United States), and 0.5 mg of heat-killed *Mycobacterium tuberculosis* (Difco Laboratories, Detroit, MI) in the nuchal area. Pertussis toxin per animal (300 ng, Sigma-Aldrich) was intraperitoneally injected immediately and 24 h later in EAE animals (Phillips et al., 2007).

C16 peptide (synthesized by Shanghai ZiYu Biological Technology Co., Ltd., Shanghai, China) was dissolved in distilled water with 0.3% acetic acid. The peptide solution was sterilized by filtration through a 0.22-μm disc filter and neutralized to pH 7.4 with sterilized NaOH. An equal volume of sterile phosphate-buffered saline was added to buffer the solution, resulting in a final concentration of 2 mg/mL. The vehicle group was prepared following the same procedure without adding the peptide. The normal control group was not treated, while other groups received the following treatments: The vehicle group received 1 mL of the C16-free solvent. The C16 group received C16 alone (1 mg/kg/day). The C16+Tie2 inhibitor group received a mixture of C16 (1 mg/kg/day) and Tie2-kinase inhibitor (25 mg/kg; Cat No. S1577, Selleck Chem., Houston, TX, United States). The C16+LY294002 group received a mixture of C16 (1 mg/kg/day) and PI3K/Akt inhibitor LY294002 (100 mg/kg; Cat No. S1105, Selleck Chem.) (Chen et al., 2019). All drugs were administrated via intravenous injection through the tail vein. The initial dose of treatments was given immediately after EAE induction. Then, the solutions were injected intravenously each day for 2 weeks.

Beginning on day seven after EAE induction, the body weight of the rats was measured daily, and disease severity was assessed using the clinic score until the end of the experiment: 0, no signs; 1, partial loss of tail tonicity; 2, loss of tail tonicity; 3, unsteady gait and mild paralysis; 4, hind limb paralysis and incontinence; 5, moribund or death (Cai et al., 2021). The success of the EAE model was defined as a score over 2.

The study was approved by the animal ethical committee of Zhejiang University (approval number: 13074), and all experiments were performed according to the National Institute of Health Guide for the Care and Use of Laboratory Animals.

### 2.2 Neurophysiological assessment

At the 2 (the peak stage of acute EAE model) weeks post-immunization, the neurophysiological assessment was performed by recording the cortical somatosensory evoked potentials (c-SEPs) and cortical motor evoked potentials (c-MEPs) of five rats from each group at 2 weeks after EAE induction just before sacrifice according to a previous study (Zhang et al., 2014).

### 2.3 Enzyme-linked immunosorbent assay (ELISA) and reactive oxygen species (ROS) measurement

The peripheral blood samples of rats (n = 5) were collected at 2 weeks (the peak stage of the acute EAE model), and the plasma samples were incubated in 96-well plates precoated with anti-interleukin (IL)-1β (Cat No. SRLB00; 1:400, R&D Systems, Minneapolis, MN, United States), anti-IL-10 (Cat No. R1000; 1:400, R&D Systems) and anti-TNF-α (Cat No. RTA00; 1:200, R&D Systems) antibodies. ELISA was performed following the protocol.

To detect the levels of ROS in the serum, blood samples were centrifuged for 5 min at 3,000 rpm. ROS were detected using dichlorofluorescein-diacetate (DCFH) (Cat No. D6665, Merck Sigma-Aldrich, Shanghai, China). A volume of 12 µL serum was incubated with 1,000 µL of TE buffer, 10 µL of NaOH, and 10 µL of DCFH solution for 10 min. The fluorescence signal was detected after creating an emission spectrum between 500 and 550 nm, with the emission peak at 525 nm recorded.

### 2.4 Preparation of tissues for histology assessment

At 2 and 4 weeks post-immunization under deep anesthesia with sodium pentobarbital (40 mg/kg), the rats were perfused with cold saline followed by 4% paraformaldehyde in 0.1 M phosphate buffer (PB, pH 7.4) via the left cardiac ventricle. Following the careful dissection of the spinal cord and brain, the lumbar segment of the spinal cord at 1 cm in length and one hemisphere of the brain of each animal were fixed in 4% paraformaldehyde in 0.1 M PB (pH 7.4) for 4 h and dehydrated in 30% sucrose in phosphate-buffered saline (PBS) until the tissue sank to the bottom of the container. The tissues were cut into 20 µm coronal sections for the brain hemisphere and transverse sections for the spinal cord with a Leica cryostat (Cat No. CM1860, Leica Biosystem, IL, United States), and the sections were mounted onto slides precoated with 0.02% poly-L-lysine, for histological assessment and immunohistological and immunofluorescent staining. The remaining part of the spinal cord and another brain hemisphere were fixed in a 5% glutaraldehyde solution for the transmission electron microscope.

### 2.5 Histological assessment

The neuron survival and inflammation were evaluated with Cresyl violet (Nissl) staining. These neurons, having a nucleus with a clear boundary and a soma with sufficient endoplasmic reticulum, were searched and accounted for as survival neurons. The evaluation of the severity degree of inflammatory infiltration was conducted according to the inflammatory score by a previous study (Fang et al., 2013): 0, no detected inflammatory cell; 1, inflammatory cell infiltration detected only around blood vessels and meninges; 2, mild inflammatory cell infiltration detected in the parenchyma (1–10 cells/section); 3, moderate inflammatory cell infiltration detected in the parenchyma (11–100 cells/section); and 4, serious inflammatory cell infiltration in the parenchyma (>100 cells/section).

According to a previous study, the demyelination in the white matter tract was examined with a modified eriochrome cyanine staining (ECR) protocol (Jiang et al., 2014). Demyelination degree was evaluated according to the following criteria described in (Fang et al., 2013): grade 0, normal white matter without myelin damage; grade 1, rare loci of demyelination; grade 2, scanty demyelination in white matter; grade 3, affluent demyelination in perivascular or subpial regions; grade 4, heavy perivascular and subpial demyelination with leukocyte infiltration in 50% of the spinal cord parenchyma; and grade 5, extensive perivascular and subpial demyelination with leukocyte infiltration in the entire spinal cord parenchyma.

Bielschowsky silver staining was performed as described previously to estimate axonal loss (Fang et al., 2013). The axonal loss was assessed using the following scale (Yin et al., 2010): 0, no axonal loss; 1, a few foci of superficial axonal loss involving less than 25% of the tissue; 2, foci of deep axonal loss that encompassed over 25% of the tissue; and 3, diffuse and widespread axonal loss.

### 2.6 Immunofluorescence staining

A water-repellent pap pen (Cat No. 008877; Invitrogen, Carlsbad, CA, United States) was used to apply a wax ring around fixed tissue sections. Then, tissues were rinsed with 0.01 M Tris-buffered saline for 10 min. Permeabilization and blocking were subsequently performed with 0.3% Triton X-100/10% normal goat serum in 0.01 M PBS for 30 min before the sections were incubated with the following primary antibodies overnight at 4°C: anti-CD206 (1:500; Cat No. Ab64693, Abcam, Cambridge, MA, United States), anti-pTie-2 (1:200; Cat No. OM126899; OmnimAbs, Alhambra, CA, United States), anti-zonula occludens 1 antibody (ZO-1; 1:200; Cat No. sc-33725, Santa Cruz Biotechnology, United States), anti-AKT (1:1,000; Cat No. GTX28805, GeneTex Inc., Irvine, CA, United States), anti-caspase 3 (1:500; Cat No. sc-56053, Santa Cruz Biotechnology, United States), anti-160KD neurofilament M (NF-M; 1:1,000; Cat No. ab134458, Abcam), anti-mouse anti-myelin basic protein (MBP; 1:500; Cat No. ab11159, Abcam), anti-CD86 (1:1,000; Cat No. ab238468, Abcam), anti-RHO (1:400; Cat No. 1B3-4A10, Thermo Fisher Scientific, Waltham, MA, United States), anti-αvβ3 (1:800; Cat No. BMB5, Millipore), and anti-α5β1 (1:800; Cat No. M200; Enzo Biochem, Inc. Farmingdale, NY, United States). Then, the blots were briefly rinsed in PBS and incubated with goat anti-rabbit IgG secondary antibodies conjugated with FITC (1:200; Cat No. sc-2010, sc-2012, sc-2780, Santa Cruz Biotechnology, United States) for 1 h at 37°C. The sections were then placed on slides with anti-fade Gel/Mount aqueous mounting media (Cat No. ab128982, Abcam). In the control sections, the primary antibodies were omitted.

### 2.7 Evaluation of brain/spinal-blood-barrier disruption

Rats (n = 3 per group) were randomly chosen to assess blood-brain barrier (BBB) permeability using a modified Evans blue extravasation method. Briefly, rats were anesthetized via intraperitoneal administration of 40 mg/kg sodium pentobarbital and infused with 37°C Evans blue dye (2% in 0.9% normal saline, 4 mL/kg) through the right femoral vein over 5 min. Two hours later, rats were perfused with 300 mL of normal saline to remove any residual dye in the blood vessels. BBB permeability was then evaluated in the motor cortex and spinal cord tissues. Half of the tissues were removed, isolated, and mechanically homogenized in 750 µL of N,N-dimethylformamide (Cat.No 68-12-2, Merck Sigma-Aldrich, United States). The obtained suspension was kept at room temperature in the dark for 72 h and then centrifuged at 10,000 × g for 25 min. The supernatant was analyzed using a spectrophotometer (Molecular Devices OptiMax M5/M5e, United States) at 610 nm. Dye concentrations were expressed as µg/g of tissue weight and calculated from a standard curve obtained using known amounts of the dye.

### 2.8 Histological examination with transmission electron microscopy (TEM)

As described previously, tissues were processed for EM examination (Jiang et al., 2014). EM images of various parts of the brain cortex and lumbar spinal cord were captured.

### 2.9 Western blotting

Western blotting assay was performed using the following antibodies: polyclonal rabbit anti-CD206 (1:1,500; Cat No. ab64693, Abcam), anti-pTie-2 (1:500; Cat No. OM126899; OmnimAbs), anti-zonula occludens 1 (ZO-1) (1:1,500; Cat No. sc-33725, Santa Cruz Biotechnology), anti-AKT (1:2,000; Cat No. GTX28805; GeneTex Inc.), anti-caspase-3 (1:1,500; Cat No. sc-56053, Santa Cruz Biotechnology, United States), anti-NF-M (1:1,500; Cat No. ab134458, Abcam), mouse anti-myelin basic protein (MBP; 1:1,500; Cat No. ab11159, Abcam), anti-CD86 (1:1,500; Cat No. ab238468; Abcam), and anti-RHOA (1:400; Cat No. 1B3-4A10, Thermo Fisher Scientific). The protein bands were normalized with β-actin (1:5,000; Cat No. MABT825, Merck Sigma-Aldrich). In the negative control experiments, the primary antibodies were replaced with PBS.

### 2.10 Statistical analysis

Differences in protein levels among different groups were assessed using two-way analysis of variance (ANOVA) followed by Tukey’s post-hoc test. Kruskal-Wallis nonparametric one-way analysis of variance (ANOVA) was used for data presented as percentages, followed by *post hoc* analysis. Histological scores were compared using the Mann-Whitney U test. Continuous data were presented as mean ± standard error (SEM). All data were analyzed using SPSS 13.0 software, and *p*-values less than 0.05 were considered statistically significant. Graphical representation of statistical data was created using GraphPad Prism Version 5.0 (GraphPad Prism Software, Inc., San Diego, CA, United States).

## 3 Results

### 3.1 C16 peptide binds to αvβ3 and α5β1

Double immunofluorescence staining of αvβ3/α5β1 (red) and cell surface markers showed that cells expressing αvβ3 and α5β1 under EAE or EAE plus other treatments were mainly endothelial cells and leucocytes (Supplementary Figure S1). Moreover, co-localization of FITC-conjugated C16 peptide (green) and integrins αvβ3 (red, Supplementary Figures S2A–D) and α5β1 (red, Supplementary Figures S2E–H) showed that C16 peptide that could bind to integrins αvβ3 *and* α5β1.

### 3.2 C16 peptide treatment mitigated weight loss in EAE rats

While the normal control group exhibited a steady increase in body weight over time (starting at 250 g on the first day of the study), all other groups showed a decrease in body weight. Among them, rats treated with C16 demonstrated the slowest decline in body weight compared to the other three groups (Supplementary Figure S3).

### 3.3 Tie-2 inhibitor and especially LY294002 application attenuated the protective effects of C16 on neurological function scores of EAE

**FIGURE 1:**
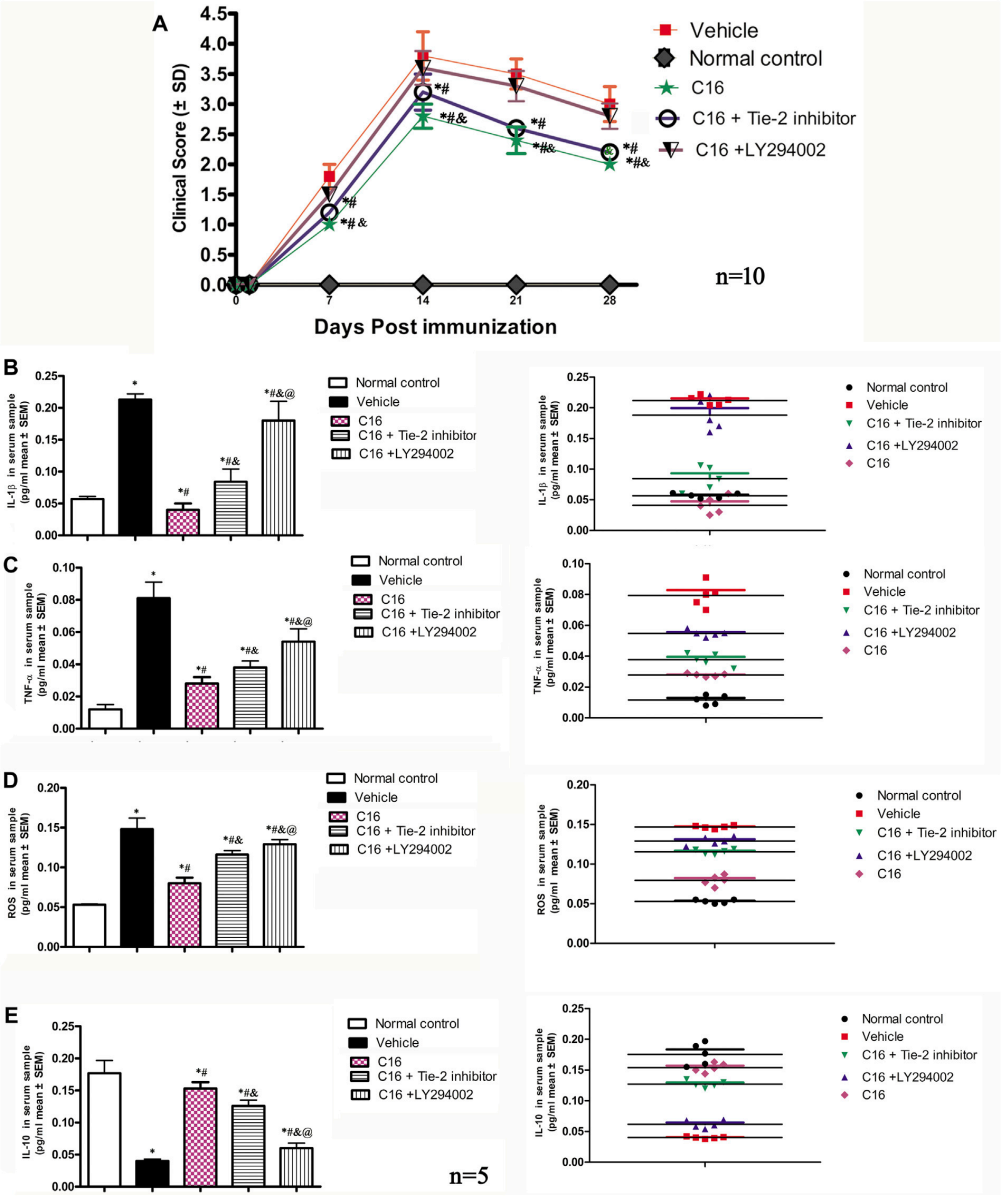
**(A)** C16 and C16+Tie-2 inhibitor groups could delay the onset of motor-related symptoms and reduce the disease severity in EAE rats both in the peak stage (2 weeks post-immunization) and the self-recovery stage (4 weeks post-immunization), when compared with the vehicle and C16+ LY294002 groups. However, compared with the C16-treated group, the C16+Tie-2 inhibitor application impaired its improvement in the clinical scores of rats. n = 10 per group.*p < 0.05 vs. vehicle group. ^#^ *p* < 0.01 vs. C16+ LY294002 group. ^&^ *p* < 0.05 vs. C16+Tie-2 inhibitor. **(B–E)** Effects of C16, C16+Tie-2 inhibitor, and C16+PI3K inhibitor on the serum concentration of **(B)** IL-1β, **(C)** TNF-a, **(D)** ROS, and **(E)** IL-10 measured by ELISA. n = 5 per group. (Left, box plot; Right, dot plot). *, *p* < 0.05 vs. normal control group; #, *p* < 0.05 vs. vehicle group; &, *p* < 0.05 vs. C16 group; @, *p* < 0.05 vs. C16+Tie-2 inhibitor group. Differences between different groups were analyzed using two-way ANOVA, followed by Tukey’s post-hoc test.

The clinical function scores indicated that MS symptoms appeared 7-8 d after immunization (clinical score < 2) in the vehicle group. These rats rapidly developed to the acute stage (clinical score 3-4) and peaked at 2 weeks after immunization (Figure 1A). The clinical score both in the peak stage (2 weeks post-immunization) and the self-recovery stage (4 weeks post-immunization) of the vehicle group was significantly higher when compared to that of the C16 group and C16+Tie-2 inhibitor group (all *p* < 0.05), but not the C16+LY294002 group. The C16 group demonstrated a significant improvement in function compared to the vehicle group and C16+LY294002 group. However, Tie-2 inhibitor and especially LY294002 treatment decreased the effects of C16 (*p* < 0.05, Figure 1A).

Furthermore, rats in the vehicle group showed significantly longer latency and smaller amplitudes in both c-SEP and c-MEP recordings than those in the normal control group (Supplementary Table S1), suggesting reduced nerve conduction speed and the number of surviving fibers. Treatment with C16 and C16+Tie-2 inhibitors significantly mitigated these changes (all *p* < 0.05). However, the C16+LY294002 group did not significantly improve compared to the C16 and C16+Tie-2 inhibitor groups (*p* < 0.05).

### 3.4 PI3K inhibitor LY294002 demonstrated stronger inhibition than Tie-2 inhibitor on the attenuation of inflammation by C16

**FIGURE 2:**
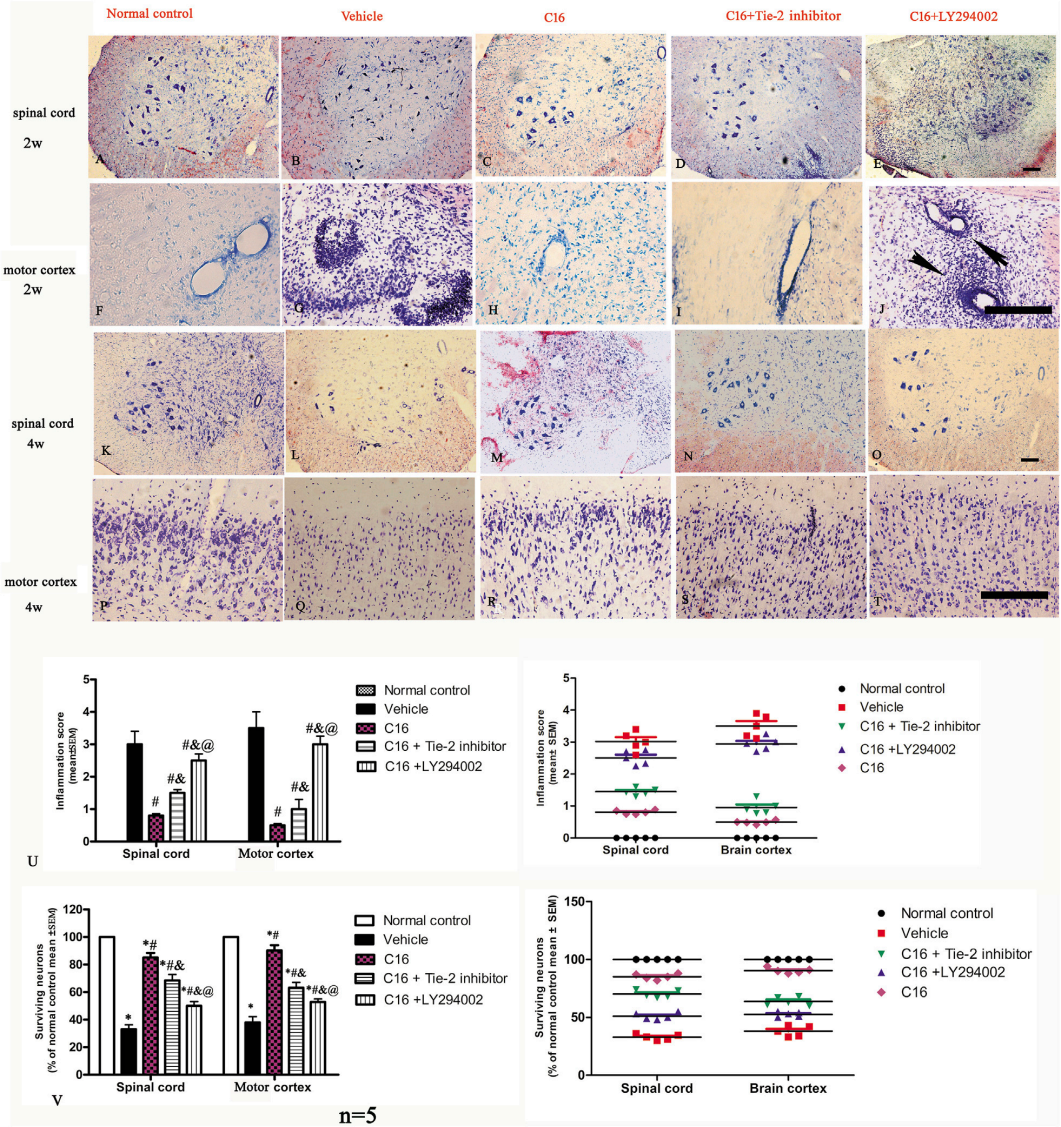
Tie-2 inhibitor and PI3K inhibitor attenuated the suppression of inflammation score induced by C16 in the EAE model. **(A–J)** Representative images by Nissl staining to show the effects of C16, C16+Tie-2 inhibitor, and C16+PI3K inhibitor LY294002 on cellular infiltration in the brain cortex and spinal cord at 2 weeks (the peak stage of inflammation) post-immunization. The black arrows in J showed the typical blood vessel cuffing of inflammatory cell infiltration of EAE. **(K–T)** Representative images of staining with survival neurons at 4 weeks (self-recovery stage of acute EAE). **(U)** Quantitative analysis of inflammatory scores. (Left, box plot; Right, dot plot). n = 5 per group. #, *p* < 0.05 vs. vehicle group; &, *p* < 0.05 vs. C16 group; @, *p* < 0.05 vs. C16+Tie-2 inhibitor group. **(V)** Quantitative analysis of survival neurons in the spinal cord and motor cortex of different groups. (Left, box plot; Right, dot plot). n = 5 per group. *, *p* < 0.05 vs. normal control group; #, *p* < 0.05 vs. vehicle group; &, *p* < 0.05 vs. C16 group; @, *p* < 0.05 vs. C16+Tie-2 inhibitor group. Tissues from the spinal cord and motor cortex were used for imaging analysis. Scale bar = 100 μm. Kruskal-Wallis nonparametric one-way ANOVA followed by *post hoc* analysis was used to compare data presented as percentages. Differences in the histological score between different groups were analyzed using the Mann-Whitney U test.

Nissl staining show visible infiltration of inflammatory cells in the brain cortex and spinal cord at 2 weeks (the peak stage of inflammation) post-immunization in vehicle group. The results indicated that the inflammatory score was significantly higher in the CNS of rats in the vehicle group and was attenuated by C16 treatment. However, the Tie-2 inhibitor, especially the PI3K inhibitor LY294002, remarkably impaired the effects of C16 in attenuating CNS inflammation (Figures 2A–J, U).

When compared to the normal control group, the serum levels of IL-1β, tumor necrosis factor-alpha (TNF-α), and ROS in the vehicle group were increased with significant differences (*p* < 0.05). The increase in the IL-1β, TNF-α, and ROS levels was attenuated by treatments with C16 (Figures 1B–D). In contrast, the serum level of IL-10, an anti-inflammatory factor, significantly decreased in the vehicle group compared to the normal control group. This downregulation was reversed by treatment with C16 (Figure 1E). However, co-treatment of C16 with Tie-2 inhibitor or PI3K inhibitor LY294002 significantly attenuated the effects of C16. The impairment effect of LY294002 was more serious than the Tie-2 inhibitor group (Figure 1).

### 3.5 The effects of C16 in alleviating demyelination and axon damage induced by EAE was impaired by Tie-2 inhibitor and especially by PI3K inhibitor LY294002

**FIGURE 3:**
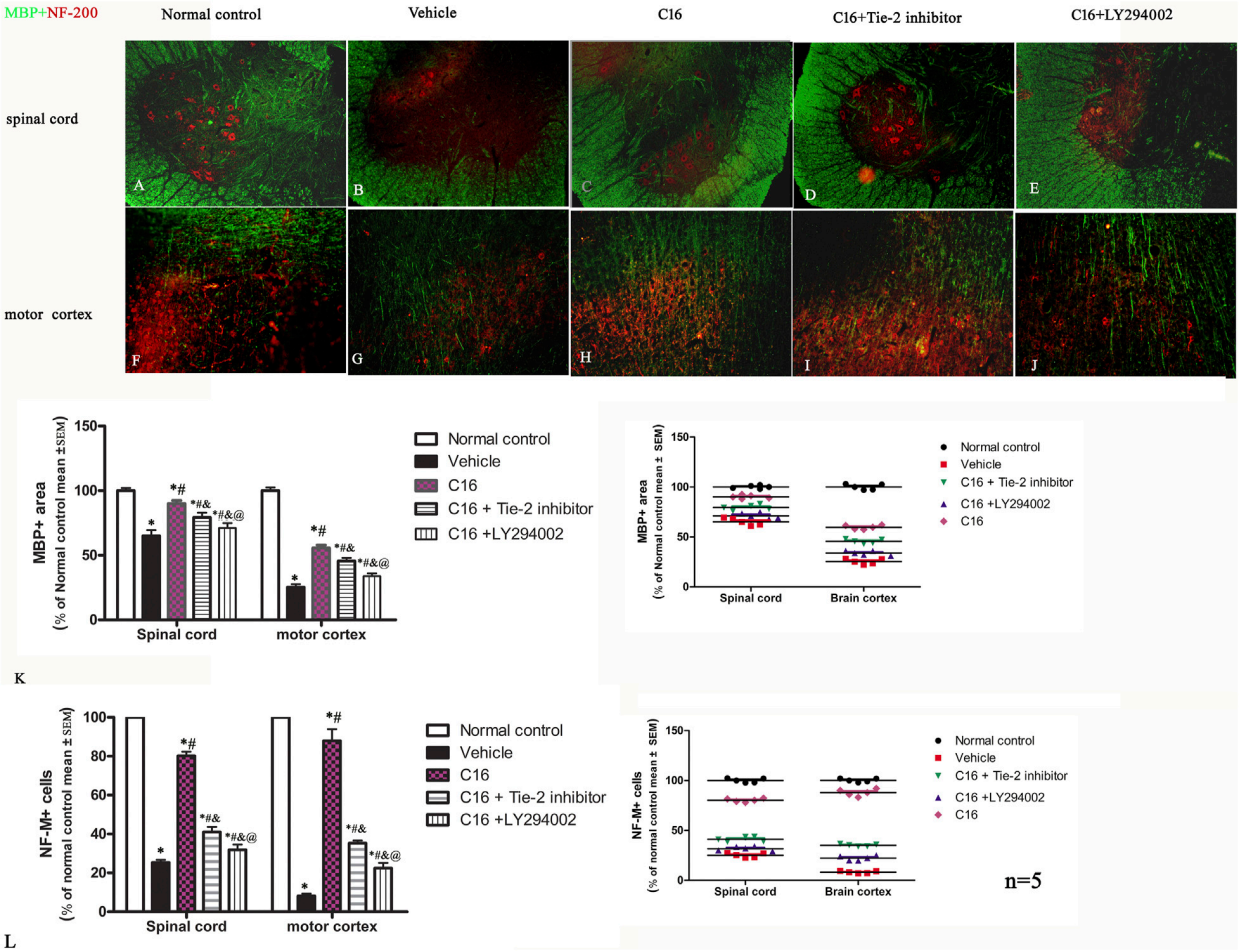
Effects of C16, C16+Tie-2 inhibitor, and C16+PI3K inhibitor on demyelination induced by EAE. **(A–J)** Representative images of MBP (green) and neurofilament neurofilaments (NF-M, red) double immunostaining. **(K–L)** Quantification of **(K)** MBP-labeled cells and **(L)** NF-M-labeled cells in different groups. (Left, box plot; Right, dot plot). n = 5 per group. *, *p* < 0.05 vs. normal control group; #, *p* < 0.05 vs. vehicle group; &, *p* < 0.05 vs. C16 group; @, *p* < 0.05 vs. C16+Tie-2 inhibitor group. Tissues from the spinal cord and motor cortex were used for imaging analysis. Scale bar = 100 μm. Kruskal-Wallis nonparametric one-way ANOVA followed by *post hoc* analysis was used to compare data presented as percentages.

The immunohistochemical labeling with MBP (Figure 3 green) and ECR (Supplementary Figure S4) staining for myelination indicated that the vehicle group had large areas of demyelination compared to the normal control group rats. Treatment with C16 significantly kept the myelination (Figure 3K), reduced demyelination areas, and improved demyelination scores (Supplementary Figure S4I). Tie-2 inhibitor and PI3K/Akt inhibitor significantly attenuated the effect of C16 (Figure 3K; Supplementary Figure S4I).

NF-M (Figure 3, red) and Bielschowsky staining (Supplementary Figure S4) exhibited severe axon loss in the vehicle group. Compared with the vehicle group, changes in the axons and neurons in the spinal cord and brain were significantly improved in C16 and C16+Tie-2 inhibitor groups (Figures 3A–J). Western blotting assay was per the morphologic results, showing that C16 treatment could increase the expression levels of MBP (Supplementary Figure S5A) and NF-M (Supplementary Figure S5B), as well as the axonal scores (Supplementary Figure S4II), but the beneficent effects were impaired by Tie-2 inhibitor and especially by PI3K inhibitor LY294002.

In the EAE animal model, a significant infiltration of leukocytes in the white matter and extensive demyelination were observed (Supplementary Figure S6). Compared to other EAE-induced groups, rats subjected to C16 peptide treatment exhibited a significantly larger area of myelination (Supplementary Figure S6; labeled by anti-MBP antibody, red) in both the posterior and anterior funicular regions. This was accompanied by reduced inflammatory infiltration, as indicated by CD45 staining (green, Supplementary Figure S6).

### 3.6 Tie-2 inhibitor and especially PI3K inhibitor LY294002 decreased the effects of C16 in reducing neuronal loss/apoptosis in EAE rats

**FIGURE 4:**
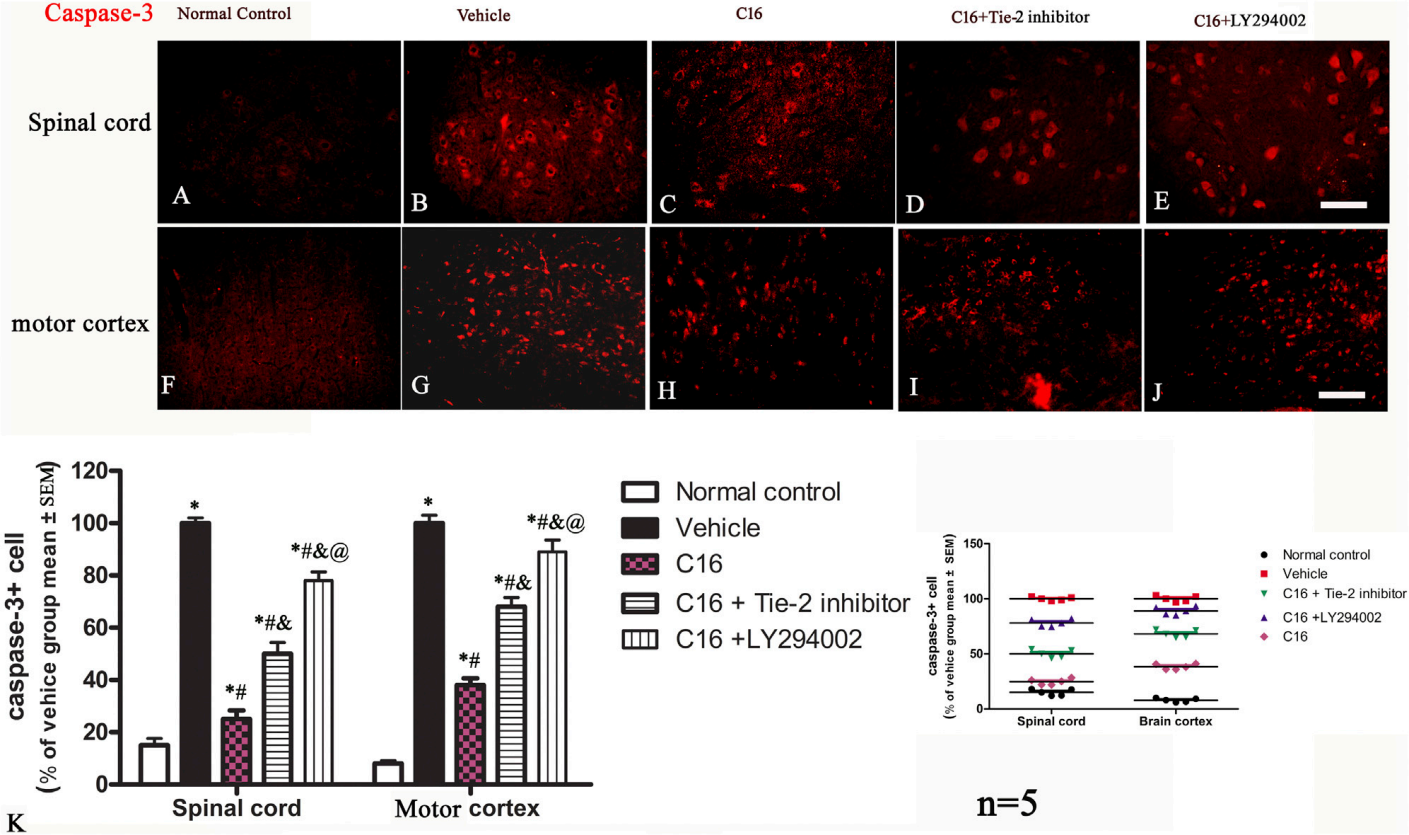
Effects of C16, C16+Tie-2 inhibitor, and C16+PI3K inhibitor on neuron apoptosis induced by EAE. **(A–E)** Representative images of the spinal cord and **(F–J)** motor cortex showing apoptotic neurons stained by caspase-3. **(K)** Quantification analysis of caspase-3-labeled cells in different groups. (Left, box plot; Right, dot plot). n = 5 per group. *, *p* < 0.05 vs. normal control group; #, *p* < 0.05 vs. vehicle group; &, *p* < 0.05 vs. C16 group; @, *p* < 0.05 vs. C16+Tie-2 inhibitor group. Tissues from the spinal cord and motor cortex were used for imaging analysis. Scale bar = 100 μm. Kruskal-Wallis nonparametric one-way ANOVA followed by *post hoc* analysis was used to compare data presented as percentages.

The neuronal loss and survival at 4 weeks pi were measured by Nissl staining (Figures 2K–T, V). The results indicated that the rats in the vehicle group exhibited visible neuron loss in the spinal cord (Figure 2L) and motor cortex (Figure 2Q) when compared with rats from the normal control group (Figures 2K, P). Compared to the vehicle group, the anterior horn of the spinal cord and the motor cortex in rats from the C16 group had a higher number of neurons (Figures 2M, R); however, the co-treatment with Tie-2 inhibitor (Figures 2N, S) and especially PI3K inhibitor LY294002 (Figures 2O, T) significantly attenuated the beneficial effects of C16 (Figure 2V). To further examine the effect of C16 on neuronal apoptosis, active caspase-3, an enzyme involved in the execution of apoptosis, was examined with immunostaining. Both pan-caspase-3 (32 kDa) and the cleaved form (19 kDa) were detected in immunofluorescence staining (Figure 4; Supplementary Figure S7) and Western blotting (Supplementary Figures S5C, H). The results indicated that caspase-3 was upregulated in the population of large multipolar motor neurons in the anterior horn of the spinal cord and the pyramidal motor neurons of the precentral gyrus of rats in the vehicle group (Figures 4B, G) when compared to rats in the normal control group (Figures 4A, F). The upregulation of caspase-3 was significantly reversed in the C16 group (Figures 4C, H). However, the co-treatment of Tie-2 inhibitor (Figures 4D, I) and PI3K inhibitor LY294002 (Figures 4E, J) significantly decreased the effects of C16 (Figure 4K). These results were consistent with the western blotting results (Supplementary Figures S5C, H). The results of cleaved caspase-3 (Supplementary Figure S7) and TUNEL assay also revealed that the C16 peptide inhibited neuronal apoptosis (Supplementary Figure S7).

**FIGURE 5:**
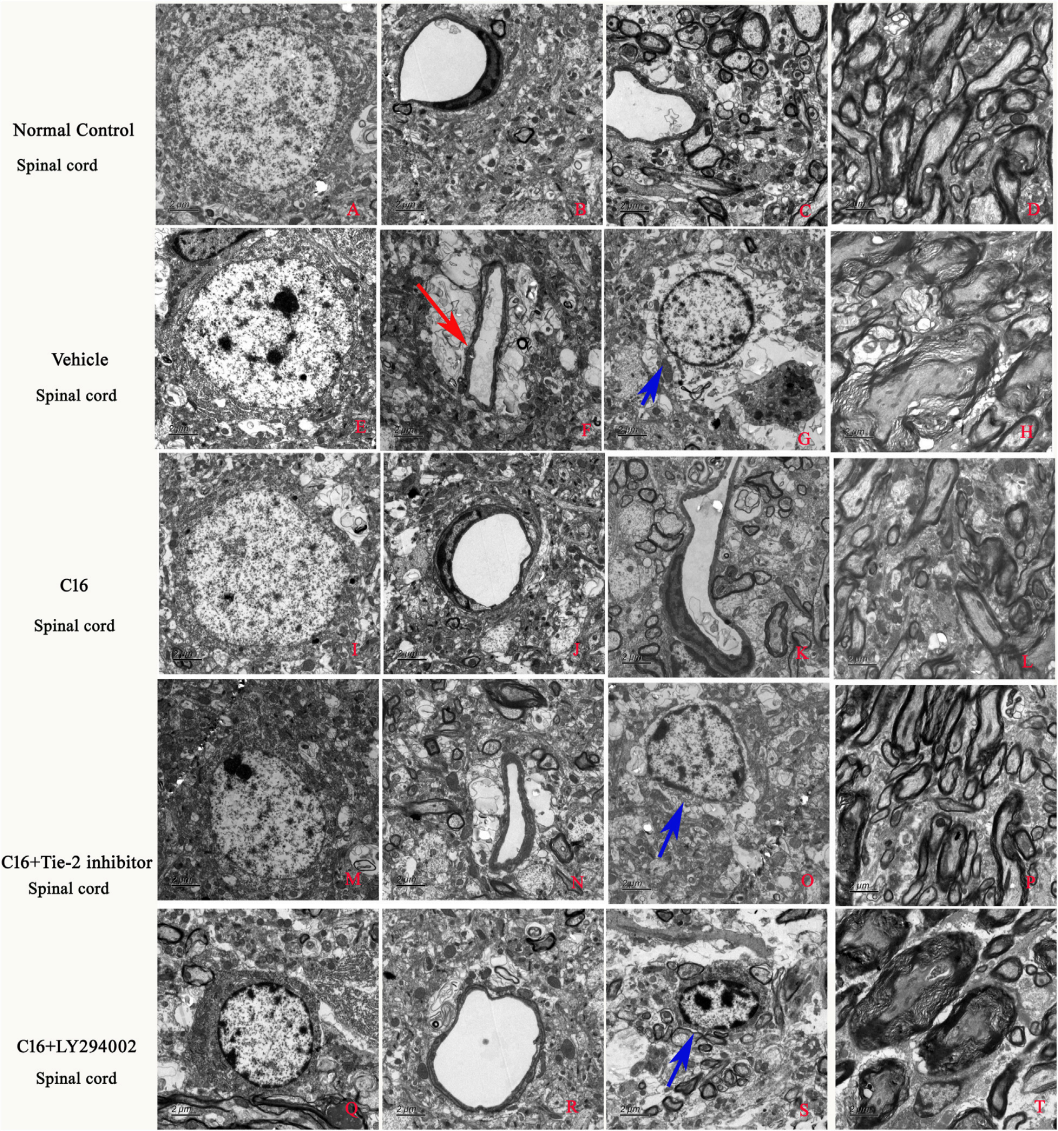
Effects of C16, C16+Tie-2 inhibitor, and C16+PI3K inhibitor on demyelination induced by EAE. **(A)** The examination with TEM further revealed that in the normal control group, the normal neurons have non-condensed chromatin in the nuclei, which is termed euchromatin and stains less deeply than heterochromatin. Normal tissues without **(B)** edema or blood vessel leakage, **(C)** inflammation, or **(D)** normal myelinated axons with dark ring-shaped myelin sheaths surrounding the axon. In the vehicle group, the neurons showed **(E)** shrinking of nuclei with condensed, fragmented, and marginated chromatin. **(H)** Many myelin sheaths show loosened, wobbly, and unfastened changes indicating demyelination. Axons shrunk or were masked by disrupted myelin sheaths. In addition, **(F)** severe edema (red arrow) and **(G)** emigration of inflammatory cells out of the blood vessels in the perivascular region (blue arrow) led to enlarged perivascular spaces. In the C16 group, the changes in **(I)** nuclei morphology, **(J)** blood vessels, (L) myelin and axons, and **(K)** tissue edema/inflammation were significantly alleviated. All these perivascular spaces were much smaller than those in the vehicle group, with compressed, solid myelin. In the **(M–P)** C16+Tie-2 inhibitor and especially the **(Q–T)** C16+PI3K inhibitor LY294002 group, the protective effects in **(O, S)** tissue edema/inflammatory cell infiltration (blue arrows), blood vessels, myelin and axons, and inflammatory cell infiltration by C16 were significantly attenuated. The ultrastructural morphology more closely resembled the vehicle control cells. Tissues from the spinal cord were used for imaging analysis. Scale bar = 2 μm. n = 5 per group.

The examination with TEM of the ultrastructural morphology in nuclei, edema, or blood vessel leakage, as well as myelin and axons of each group, were in according with the immunostaining (Figure 5).

### 3.7 C16 treatment could reduce vascular leakage and improve BBB permeability induced by EAE but destroyed by Tie-2 inhibitor and especially by PI3K inhibitor LY294002

**FIGURE 6:**
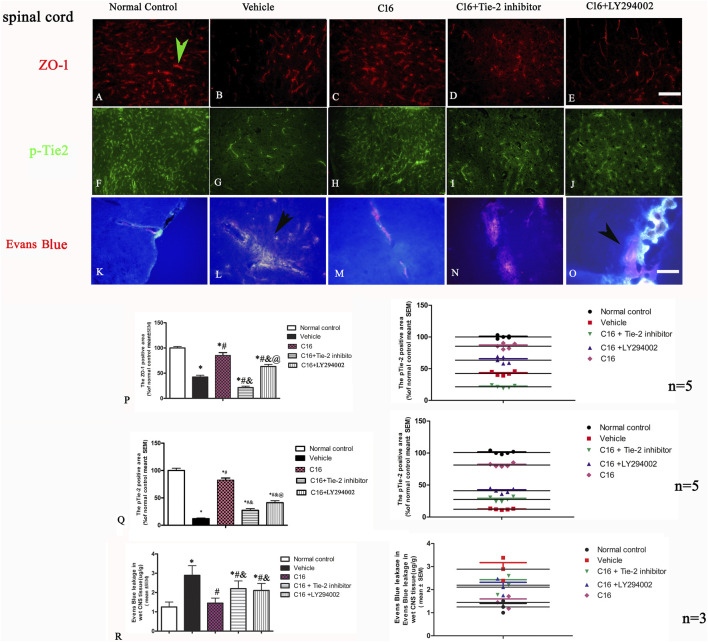
Effects of C16, C16+Tie-2 inhibitor, and C16+PI3K inhibitor on the tight junction marker ZO-1 and the Tie-2 expression. **(A–J)** Representative images of staining of the tight junction (green arrow in A) and Tie-2 expression in vascular endothelial cells (green arrow in H) by immunofluorescence staining in different groups. **(K–O)** Effects of C16, C16+Tie-2 inhibitor, and C16+PI3K inhibitor on the permeability of BBB and vascular leakage detected by Evens blue staining. It was visualized as pink-to-red color in laser excitation at 405 nm OD. Pink-to-red color indicates EB leakage due to enhanced vascular permeability. The leakage of Evens blue is indicated by arrows in L, O. Scale bar = 100 μm. **(P–Q)** Quantitative analysis of the **(P)** ZO-1 and **(Q)** Tie-2 positive areas in different groups. n = 5 per group. **(R)** Quantification analysis of EB in different groups. n = 3 per group. (Left, box plot; Right, dot plot) *, *p* < 0.05 vs. normal control group; #, *p* < 0.05 vs. vehicle group; &, *p* < 0.05 vs. C16 group; @, *p* < 0.05 vs. C16+Tie-2 inhibitor group. Tissues from the spinal cord were used for imaging analysis. Kruskal-Wallis nonparametric one-way ANOVA followed by *post hoc* analysis was used to compare data presented as percentages. Differences in the EB levels among different groups were analyzed using two-way ANOVA, followed by Tukey’s post-hoc test.

The results of TEM results indicated that the edema surrounding the blood vessels was attenuated by treatment with C16 treatment (Figure 5). Consistently, the tight junctions (immunofluorescence labeling of ZO-1) (Figures 6 A–E) between endothelial cells (ECs) were less in EAE rats of the vehicle group (Figure 6B) but were maintained in the C16 group (Figure 6C). Co-treat with Tie-2 inhibitor and PI3K inhibitor LY294002 attenuated the effects of C16 (Figure 6P). Consistently, the expression of ZO-1 protein by Western blotting showed consistent changes in different groups, i.e., ZO-1 was decreased in the vehicle group when compared to the normal control group, while C16 alleviated the decrease of ZO-1, and Tie-2 inhibitor, and PI3K inhibitor attenuated the effect of C16 (Supplementary Figure S5E).

Western blotting assay showed that treatment with Tie-2 inhibitor downregulated the expression of Tie-2 (Supplementary Figure S5E). Immunofluorescence staining (Figures 6F–J, Q) and Western blotting (Supplementary Figure S5J) revealed a similar trend of pTie-2 expression.

At the early phase of EAE, the content of Evans blue in the spinal cord and brain cortex of rats from the vehicle group was 3-fold higher than that of rats from the normal control group (Figures 6K–O, R). C16 treatment significantly decreased the Evans blue extravasation (*p* < 0.05), which was attenuated by the Tie-2 inhibitor and PI3K inhibitor LY294002 (Figure 6R).

### 3.8 Tie-2 inhibitor and especially PI3K inhibitor LY294002 attenuated the suppression of M1 phenotype and promotion of M2 phenotype of microglia/macrophages by C16 in the EAE model

**FIGURE 7:**
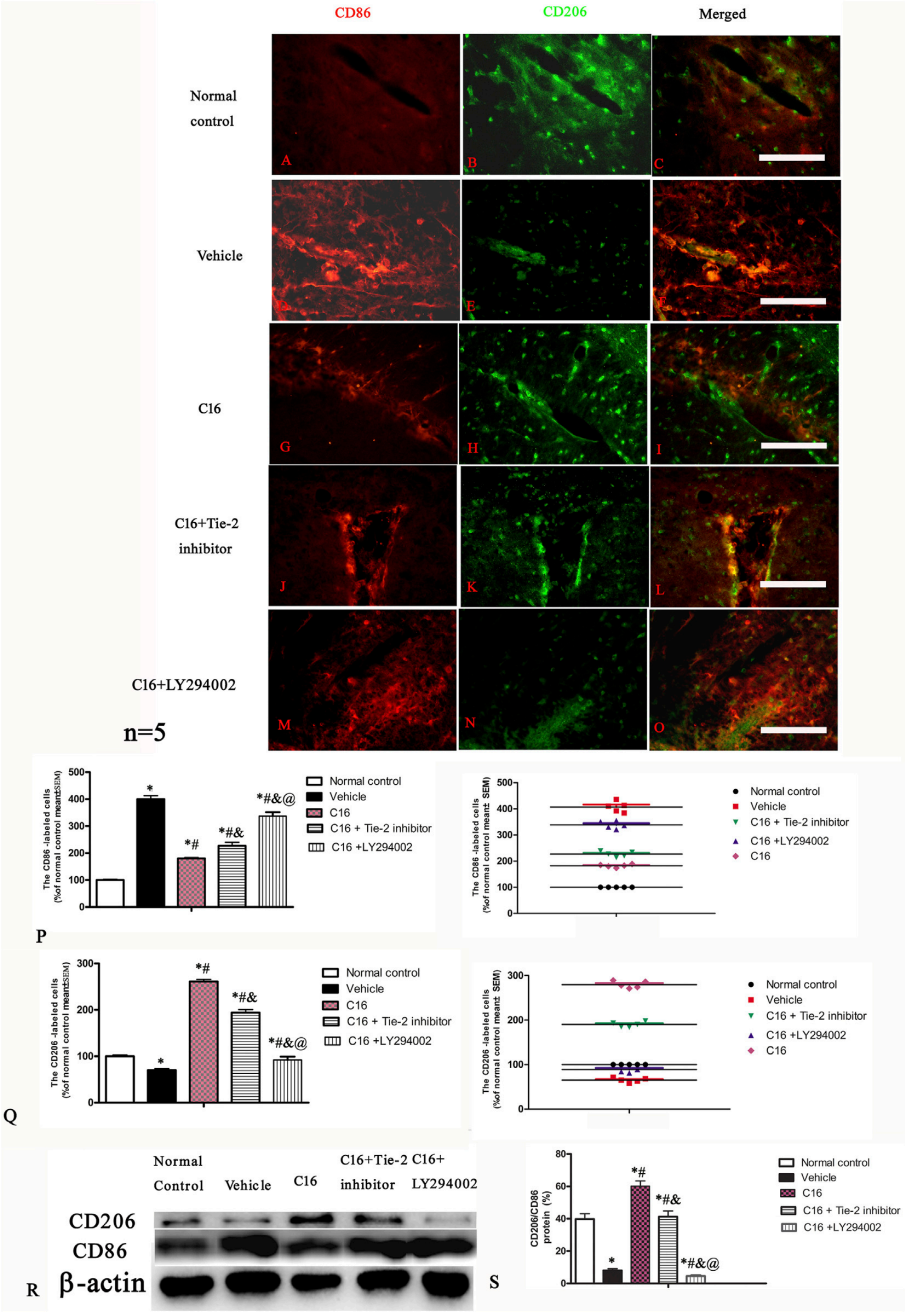
Effects of C16, C16+Tie-2 inhibitor, and C16+PI3K inhibitor on the expression of CD86 and CD206 in microglia. **(A–O)** Representative images of immunostaining of CD86 and CD206 in different groups. Tissues from the motor cortex were used for imaging analysis. **(P–Q)** Quantitative analysis of **(P)** CD86-and **(Q)** CD206-positive cells of different groups. (Left, box plot; Right, dot plot). **(R–S)** Expression of CD86 and CD206 ratio measured by Western blotting. n = 5 per group *, *p* < 0.05 vs. normal control group; #, *p* < 0.05 vs. vehicle group; &, *p* < 0.05 vs. C16 group; @, *p* < 0.05 vs. C16+Tie-2 inhibitor group. Tissues from the motor cortex were used for analysis. Scale bar = 100 μm. Kruskal-Wallis nonparametric one-way ANOVA followed by *post hoc* analysis was used to compare data presented as percentages.

Immunofluorescence staining of the spinal cord and brain tissues indicated that the expression of CD206 (M2 phenotype marker) was downregulated, while the expression of CD86 (M1 phenotype marker) was upregulated in the vehicle group when compared to the normal control group; these changes were significantly blocked by C16 (Figures 7A–O). Furthermore, the effects of C16 were significantly attenuated by Tie-2 inhibitor, especially PI3K inhibitor LY294002 (Figures 7P–Q). Western blotting showed consistent results with immunofluorescence staining results (Figures 7R, S).

### 3.9 The effects of treatment with C16, C16+Tie-2 inhibitor, and C16+LY294002 on several signaling pathways

Finally, we investigated the signaling pathway involved in the C16 effects. Western blotting showed that EAE induction (vehicle group) upregulated the expression of Rho (Supplementary Figure S5F) but inhibited the expression of AKT and p-AKT (Supplementary Figures S5G, I). These phenomena were reversed by C16 treatment. Furthermore, co-treatment of C16 with Tie-2 inhibitor, especially PI3K inhibitor LY294002 re-reversed the effect of C16 on Rho and AKT/p-AKT expression (Supplementary Figures S5F, G, I). The results of RHOA and p-AKT immunofluorescence staining were in accordance with the results of Western blotting (Supplementary Figures S8, 9).

Following EAE induction, the expression of αvβ3 and α5β1 integrins significantly increased in the vehicle group; C16 treatment further potentiated the upregulation of αvβ3 and α5β1 integrins; this upregulation was attenuated by Tie-2 inhibitor but not by PI3K inhibitor LY294002 (Supplementary Figure S10).

## 4 Discussion

Infiltration of peripheral immune/inflammatory cells through the impaired BBB can activate microglia and astrocytes by releasing inflammatory mediators. The activated microglia can produce a disastrous pro-inflammatory milieu, including stripping off myelin from axons, disruption of the BBB integrity, attraction and activation of T-lymphatic cells and monocytes, which further disassembly myelin and axon (Faissner et al., 2019; Healy et al., 2022).

In this study, we first showed that integrins αvβ3 and α5β1 are mainly expressed in endothelial cells and leucocytes, and C16 peptide could bind to both integrins. Previous studies indicated that C16 peptide showed high selectivity and affinity to αvβ3 and α5β1 integrins (Han et al., 2010; Fu et al., 2020) and could interfere with leukocytes emigrating across the endothelium and inflammatory responses by selectively binding to αvβ3 and α5β1 integrin (Zhang et al., 2014). Consistently, the present study indicated that C16 significantly increased the expressions of αvβ3, α5β1, and anti-inflammatory factor IL-10 but reduced the inflammation-related mediators, such as IL-1β, TNF-α, and ROS, thus ameliorating the inflammatory condition and improved the CNS microenvironment in EAE model. The improved micro-environment benefits neuronal survival, as evidenced by the reduction in both pan-caspase-3 and cleaved-caspase-3 expression and the decreased number of TUNEL-labeled neural cells. These results collectively suggest that neuronal damage and associated apoptosis have been mitigated.

Upregulation of Tie2, a vascular endothelial receptor tyrosine kinase that can be activated by angiopoietin-1, is associated with activating the PI3K/Akt pathway (Chen et al., 2017). Tie2 activation promotes the migration and sprouting of endothelial progenitor cells, inhibits the apoptosis of endothelial cells through Akt phosphorylation, and contributes to vascular integrity by interacting with integrins (Ye et al., 2018; Wang et al., 2019). The PI3K/Akt pathway is well-known for its role in angiogenesis (DeBusk et al., 2004). The crosstalk between Tie2 and integrins further regulates the survival and motility of injured cells (Cascone et al., 2005; Shlamkovich et al., 2018). In the present study, C16 treatment upregulated Tie-2 and pTie-2 expression. We hypothesized that C16 binds to integrins, activating Tie2 and its downstream PI3K/Akt pathway, thereby protecting blood vessels and reducing leakage. Our data showed that the beneficial effect of C16 on blood vessels could be suppressed by the Tie-2 inhibitor. The reduction of vascular permeability under inflammatory conditions and the enhancement of vessel survival are crucial because the vulnerability of the CNS to edema and ischemia can lead to demyelination, axonal damage, conduction deficits, and overt functional loss. Electrophysiological tests in this study quantified impaired nerve impulses, characterized by a reduction in amplitude (axonal damage) and latency delay (focal demyelination). Our results suggest that C16 ameliorated demyelination and axonal injury in rats, aligning with the observed effects of C16 in reducing Evans blue leakage and preserving tight junctions between endothelial cells. Previous studies also showed that increasing the expression of phosphorylated Akt could greatly diminish toxicity-induced neuron apoptosis. These neuroprotective effects were notably reversed by pretreatment with LY294002, a specific inhibitor of PI3K, suggesting that the PI3K/Akt/signaling pathway may be a possible mechanism involved in neuroprotection (Jia et al., 2014). In the present study, we found that C16 treatment promoted the phosphorylation of both AKT and Tie2. Moreover, a previous study has suggested that the Tie2/Akt/eNOS signaling pathway may serve as a potential target for vascular protection. An increase in the relative expression of pTie2/Tie2 and pAKT/AKT is considered beneficial for vascular protection (Asadian S et al., 2019). Thus, these pathways may constitute the underlying mechanisms for C16.

Our previous studies showed that modulating microglia activation might be another mechanism of C16 in protecting EAE. Microglia activation is critical in neuroinflammation and neuronal death by switching between the neurotoxic M1 phenotype and the neuroprotective M2 phenotype. M1 microglia can produce inflammatory cytokines and generate a detrimental microenvironment for neurons, while M2 microglia can produce neurotrophic factors and anti-inflammatory mediators and thus generate a supportive microenvironment for neurons. Our previous *in vitro* study (Fu et al., 2020) indicated that the C16 treatment effectively reduced the levels of CD86 (M1 phenotype markers) but elevated levels of CD206 (M2 phenotype marker) expression, suggesting C16 could change the action phenotype of microglia, which was inconsistent with the present results *in vivo*. Microglial polarization plays a vital role in the pathological process of neuroinflammation following subarachnoid hemorrhage (SAH). An increase in the levels of inflammatory mediators and the proportion of M1 cells was observed in mice following SAH induction, resulting in neuronal apoptosis at 24 h post-induction. Treatment with milk fat globule-epidermal growth factor-8 (MFG-E8) significantly reduced brain edema, improved neurological function, downregulated pro-inflammatory factors, and promoted the shift of microglial towards the M2 phenotype. However, the knockdown of MFG-E8 and integrin β3 using small interfering RNA abolished the effects of MFG-E8 on inhibiting inflammation and facilitating M2 phenotype polarization. The regulation of the integrin β3/SOCS3 (Suppressors Of Cytokine Signaling 3)/STAT3 (signal transducer and activator of transcription 3) pathway by Stattic, a STAT3 inhibitor, further elucidated the role of MFG-E8 in microglial polarization. MFG-E8 inhibits neuronal inflammation by transforming the microglial phenotype to M2 and exerts a direct protective effect on neurons after SAH, in which the integrin β3/SOCS3/STAT3 signaling pathway may be involved (Gao et al., 2021). Because integrin β3 is a receptor of the C16 peptide, it might be a potential mechanism driving microglial M2 polarization.

Furthermore, previous studies indicated that Rho could modulate the activities of PI3K and RhoA signal, change the blood vessel permeability (Overstreet et al., 2013), and switch the M1/M2 genotypes of microglia (Wang et al., 2021). The GTPase Rho controls many of the inflammatory progressions, neurodegeneration mediated by microglia activation (Scheiblich and Bicker, 2017), the preservation of normal endothelial junction (Xiao et al., 2013), axonal elongation, and axonal myelination (Xing et al., 2011). Consistent with these studies, the present results suggested that C16 could target the Rho signal to promote recovery of injured CNS in EAE rats.

Because the integrin and Tie-2 activation pathway was upstream of the PI3K/Akt pathway, their inhibition by antibodies or specific inhibitors shows less severe consequences than the LY294002 application. Similarly, since the Tie2 in the up-stream of Tie2–integrin and integrin-PI3K/Akt pathway, the upregulation of αvβ3 and α5β1 integrins induced by C16 application was attenuated by Tie-2 inhibitor but not by PI3K inhibitor LY294002.

In conclusion, the C16 peptide effectively attenuated the clinical symptoms of EAE, as evidenced by a reduction in the mean clinical score and the prevention of body weight loss. C16 treatment also significantly reduced inflammatory cell infiltration and prevented demyelination in the white matter and axon loss in neurons. Additionally, C16 suppressed the expression of inflammatory mediators, leading to a notable attenuation of vascular edema, neuronal apoptosis, and inflammatory responses in the CNS of the EAE model. All these effects may be mediated through the signaling pathways involving integrins, pAKT, Rho, pTie2/Tie2, and PI3K/AKT. These findings suggest that the C16 peptide may be a potential therapeutic target for inflammation-related CNS diseases.

## Data Availability

The raw data supporting the conclusion of this article will be made available by the authors, without undue reservation.

## Supplementary material

SUPPLEMENTARY FIGURE S1 Double staining of αvβ3/α5β1 (red) and cell surface markers (CD31, endothelial cells; CD45 leucocytes; green) showed that cells expressing αvβ3 and α5β1 under EAE and EAE plus other treatment groups are mainly endothelial cells and leucocytes. All cell nuclei were stained with Hoechst33342 nuclear dye. Scale bar = 100 μm.

SUPPLEMENTARY FIGURE S2 Co-localization (white arrows) of FITC-conjugated C16 peptide (green) and integrins αvβ3 (red, **AE–D**) and α5β1 (red, **E–H**) in the blood vessels of spinal cord. All cell nuclei were stained by Hoechst33342 nuclear dye. Scale bar=100 μm.

SUPPLEMENTARY FIGURE S3 From Day 7 after EAE induction, the daily body weight of all rats (initial weight 250 g) was monitored. In the normal control group, the average body weight steadily increased over time. In contrast, all EAE-induced groups exhibited a decline in the average body weight. The C16-treated group showed the slowest weight loss compared to the other three groups. n = 10 per group. *, p < 0.05 vs. normal control group; #, p < 0.05 vs. vehicle group. Differences among different groups were analyzed using two-way ANOVA, followed by Tukey’s post-hoc test.

SUPPLEMENTARY FIGURE S4 Effects of C16, C16+Tie-2 inhibitor, and C16+PI3K inhibitor on demyelination and axonal loss induced by EAE. **(A–O)**, Representative images of traverse sections of the lumbar spinal cord. **(A–J)** Demyelination with ECR staining. **(K–O)** Axonal loss in different groups by Bielschowsky staining. **(P–Y)** In horizontal sections of the motor cortex **(P–T)** showing the demyelination with ECR staining. **(U–Y)** showing the axonal loss in different group by Bielschowsky staining. **(I)**, Quantitative analysis of demyelination score in different groups. **(II)** Quantitative analysis of axonal loss score in different groups. (Left, box plot; Right, dot plot) n = 5 per group. #, p < 0.05 vs. vehicle group; &, p < 0.05 vs. C16 group; @, p < 0.05 vs. C16+Tie-2 inhibitor group. Tissues from the spinal cord and motor cortex were used for imaging analysis. Scale bar=100 μm. Differences in the histological score between different groups were analyzed using the Mann-Whitney U test.

SUPPLEMENTARY FIGURE S5 Effects of C16, C16+Tie-2 inhibitor and C16+PI3K inhibitor on the expression of **(A)** MBP, **(B)** NF-H, **(C)** caspase-3, **(D)** ZO-1, **(E)** RHOA, **(F)** Tie-2, **(G)** AKT, **(H)** cleaved caspase-3, **(I)** p-AKT, and **(J)** pTie-2 were assessed by Western blotting. *, p < 0.05 vs. normal control group; #, p < 0.05 vs. vehicle group; &, p < 0.05 vs. C16 group; @, p < 0.05 vs. C16+Tie-2 inhibitor group. n = 5 per group. Differences in the protein levels between different groups were analyzed using two-way ANOVA, followed by Tukey’s post-hoc test.

SUPPLEMENTARY FIGURE S6 In the EAE animal model, substantial demyelination occurred in the entire white matter, accompanied by significant leukocyte infiltration. **(A–E)** The entire spinal cord white matter in each group, with C16 peptide treatment resulting in a considerably larger myelination area (MBP, red) than in other EAE groups. **(F–J)** In the enlarged area of the posterior funiculus box shown in **A–E**, significant leukocyte infiltration (CD45, green) was observed in the vehicle and C16+LY294002 groups (white arrows), which was accompanied by notable demyelination in the white matter. **(K–O)** Double staining of MBP and CD45 showed similar trend in the anterior funiculus of each group. n = 5 per group. Scale bar = 100 μm.

SUPPLEMENTARY FIGURE S7 Effects of C16, C16+Tie-2 inhibitor, and C16+PI3K inhibitor on neuron apoptosis induced by EAE. **(A–J)** TUNEL staining showed apoptotic neurons (double stained by hematoxylin-eosin staining). **(K–T)** Immunohistochemistry staining of cleaved caspase-3 detected apoptotic neurons in each group. **(U–V)** Quantification analysis of TUNEL and cleaved caspase-3-labeled cells in different groups. (Left, box plot; Right, dot plot). n = 5 per group. *, p < 0.05 vs. normal control group; #, p < 0.05 vs. vehicle group; &, p < 0.05 vs. C16 group; @, p < 0.05 vs. C16+Tie-2 inhibitor group. Tissues from the spinal cord and motor cortex were used for imaging analysis. Scale bar = 100 μm. Kruskal-Wallis nonparametric one-way ANOVA followed by post hoc analysis was used to analyze data presented as percentages.

SUPPLEMENTARY FIGURE S8 Effects of C16, C16+Tie-2 inhibitor, and C16+PI3K inhibitor on the expression of Rho in the spinal cord and motor cortex. **(A–J)**, Representative images of the expression of RhoA shown by immunofluorescence staining both in neural cells (red arrow) and inflammatory cells (blue arrow). **(K)** Quantitative analysis of Rho-positive cells in different groups. (Left, box plot; Right, dot plot). n = 5 per group. *, p < 0.05 vs. normal control group; #, p < 0.05 vs. vehicle group; &, p < 0.05 vs. C16 group; @, p < 0.05 vs. C16+Tie-2 inhibitor group. Tissues from the spinal cord and motor cortex were used for imaging analysis. Scale bar=100 μm. Kruskal-Wallis nonparametric one-way ANOVA followed by post hoc analysis was used to analyze data presented as percentages.

SUPPLEMENTARY FIGURE S9 Effects of C16, C16+Tie-2 inhibitor, and C16+PI3K inhibitor on the expression of pAKT in the spinal cord **(A–E)** and motor cortex **(F–J)** by immunofluorescence staining. **(K)**, Quantitative analysis of the AKT-positive cells in different groups. (Left, box plot; Right, dot plot). n = 5 per group. *, p < 0.05 vs. normal control group; #, p < 0.05 vs. vehicle group; &, p < 0.05 vs. C16; @, p < 0.05 vs. C16+Tie-2 inhibitor group. Tissues from the spinal cord and motor cortex were used for imaging analysis. Scale bar =100 μm. Kruskal-Wallis nonparametric one-way ANOVA followed by post hoc analysis was used to analyze data presented as percentages.

SUPPLEMENTARY FIGURE S10 Effects of C16, C16+Tie-2 inhibitor, and C16+PI3K inhibitor on the expression of αvβ3 and α5β1 integrins. **A–L**), Representative images of αvβ3 (**A–F**, green) and α5β1 (**G–L**, green) in EC (red arrow) and inflammatory cells (white arrow). Tissues from the motor cortex were used for imaging analysis. Scale bar = 100 μm. All cell nuclei were stained with Hoechst33342 (blue). **(M–P)**, Quantitative analysis of αvβ3 and α5β1 in different groups. (Left, box plot; Right, dot plot). n = 5 per group. *, p < 0.05 vs. normal control group; #, p < 0.05 vs. vehicle group; &, p < 0.05 vs. C16; @, p < 0.05 vs. C16+Tie-2 inhibitor group. Tissues from the motor cortex were used for imaging analysis. Scale bar=100 μm.
