# Corrigendum: Neuroprotective effects of a novel peptide through the Rho-integrin-Tie2 and PI3K/Akt pathways in experimental autoimmune encephalomyelitis model

**DOI:** 10.3389/fphar.2025.1611105

**Published:** 2025-06-10

**Authors:** Wen Zhou, Han Qu, Xiao-Xiao Fu, Miao-Miao Xu, Qiang Li, Yuan Jiang, Shu Han

**Affiliations:** ^1^ Department of Emergency Medicine, The Second Affiliated Hospital of Zhejiang University School of Medicine, Hangzhou, China; ^2^ Institute of Anatomy and Cell Biology, Medical College, Zhejiang University, Hangzhou, China; ^3^ Institute of Human Anatomy, Histology and Embryology, Basic Medical College, Zhejiang Chinese Medical University, Hangzhou, China; ^4^ Department of Rehabilitation in Traditional Chinese Medicine, The Second Affiliated Hospital of Zhejiang University School of Medicine, Hangzhou, China; ^5^ Department of Pulmonology, Children’s Hospital, Zhejiang University School of Medicine, National Clinical Research Center for Child Health, Hangzhou, China

**Keywords:** C16, EAE, Tie2, integrin, PI3K/Akt pathways

In the published article, there was an error in **Supplementary Figure S7** as published. The magnification of **Supplementary Figure S7S** is different from others. This has been corrected.

The authors apologize for this error and state that this does not change the scientific conclusions of the article in any way. The original article has been updated.

