# Supplementary material for: Neuroprotective effects of a novel peptide through the Rho-integrin-Tie2 and PI3K/Akt pathways in experimental autoimmune encephalomyelitis model
Source: Front Pharmacol. 2024 Feb 6;15:1290128. doi: 10.3389/fphar.2024.1290128 (PMC10880193; doi:10.3389/fphar.2024.1290128)
Supplement: Supplementary file 1 [file Table1.DOCX]

**Supplementary Table 1.** Tie-2 inhibitor and especially LY294002 attenuated the protective effect of C16 treatment (n=10 per group)

| Group | Normal control | Vehicle | C16 | C16+Tie-2 inhibitor | C16+LY294002 |
| --- | --- | --- | --- | --- | --- |
| c-SEP latency (ms) | N 16.9±1.2 | 26.55±0.8^*^ | 18.22±0.24^*#^ | 22.39±1.23^*#&^ | 25.99±0.97^*&@^ |
|  | P 19.8+0.7 | 37.22±0.1^*^ | 22.22±0.52^*#^ | 29.47±1.34^*#&^ | 33.90±1.18^*&@^ |
| c-SEP wave amplitude  μV (mean±SD) | 27.8±1.5 | 15.8±0.8^*^ | 24.53±0.8^*#^ | 23.2±0.66^*#&^ | 13.97±0.78^*&@^ |
| c-MEP latency (ms) | 1.12±0.52 | 5.88±0.54^*^ | 2.16±0.47^*#^ | 3.34±0.24^*#&^ | 4.68±0.44^*&@^ |
| c-MEP wave amplitude  μV (mean±SD) | 12.79±0.22 | 0.3±0.02^*^ | 8.77±0.46^*#^ | 5.69±0.48^*#&^ | 1.23±0.35^*&@^ |

^*^*p*<0.05 *vs.* normal control group, ^#^*p*<0.05 *vs.* vehicle group, ^&^*p*<0.05 *vs.* LY294002 group, ^@^*p*<0.05 *vs.* C16+Tie-2 inhibitor group at the same time point.
