# Supplementary figures and images for: Neuroprotective effects of a novel peptide through the Rho-integrin-Tie2 and PI3K/Akt pathways in experimental autoimmune encephalomyelitis model
Source: Front Pharmacol. 2024 Feb 6;15:1290128. doi: 10.3389/fphar.2024.1290128 (PMC10880193; doi:10.3389/fphar.2024.1290128)

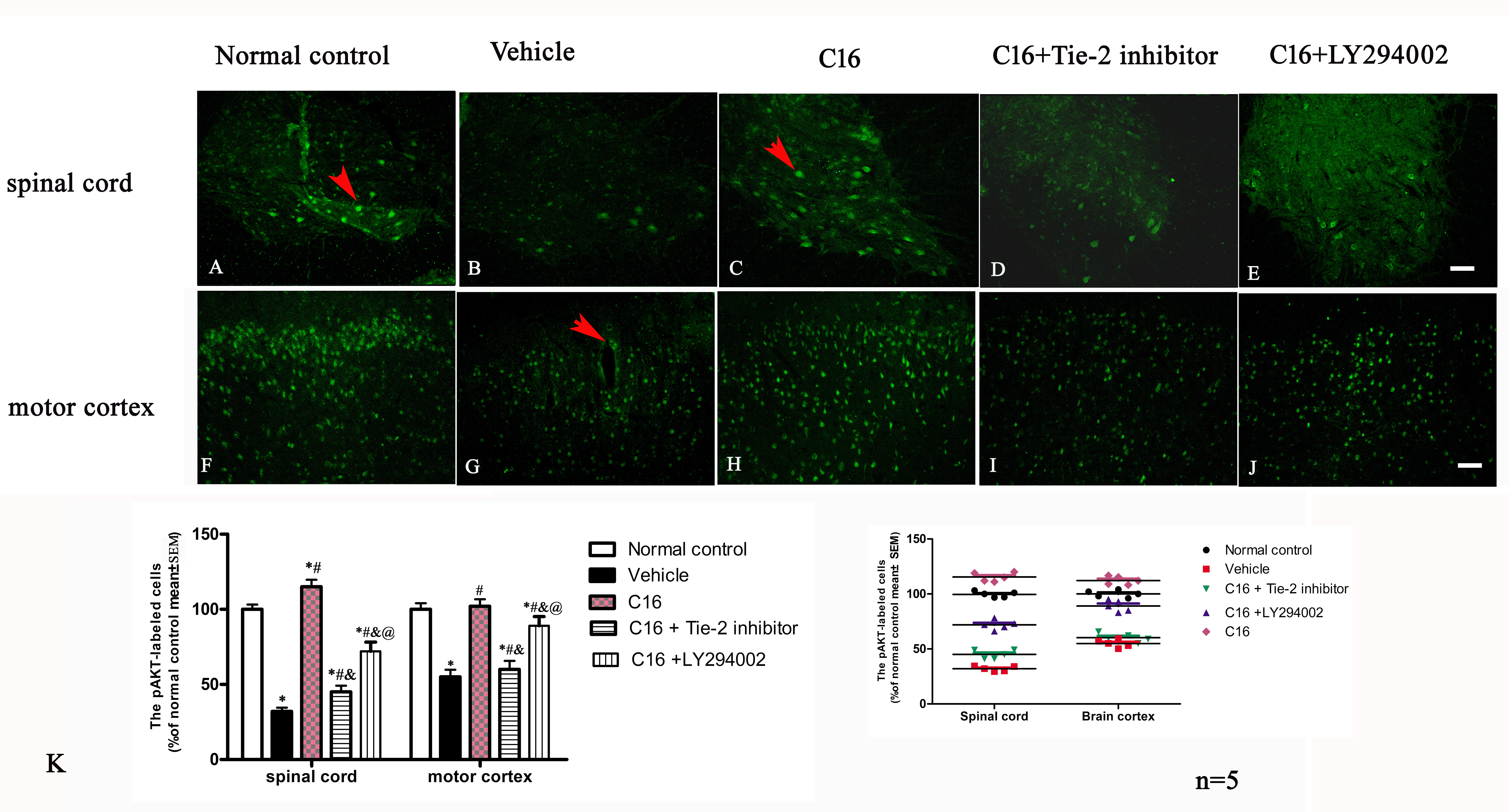

Supplement: Supplementary file 2 [file Image9.JPEG]

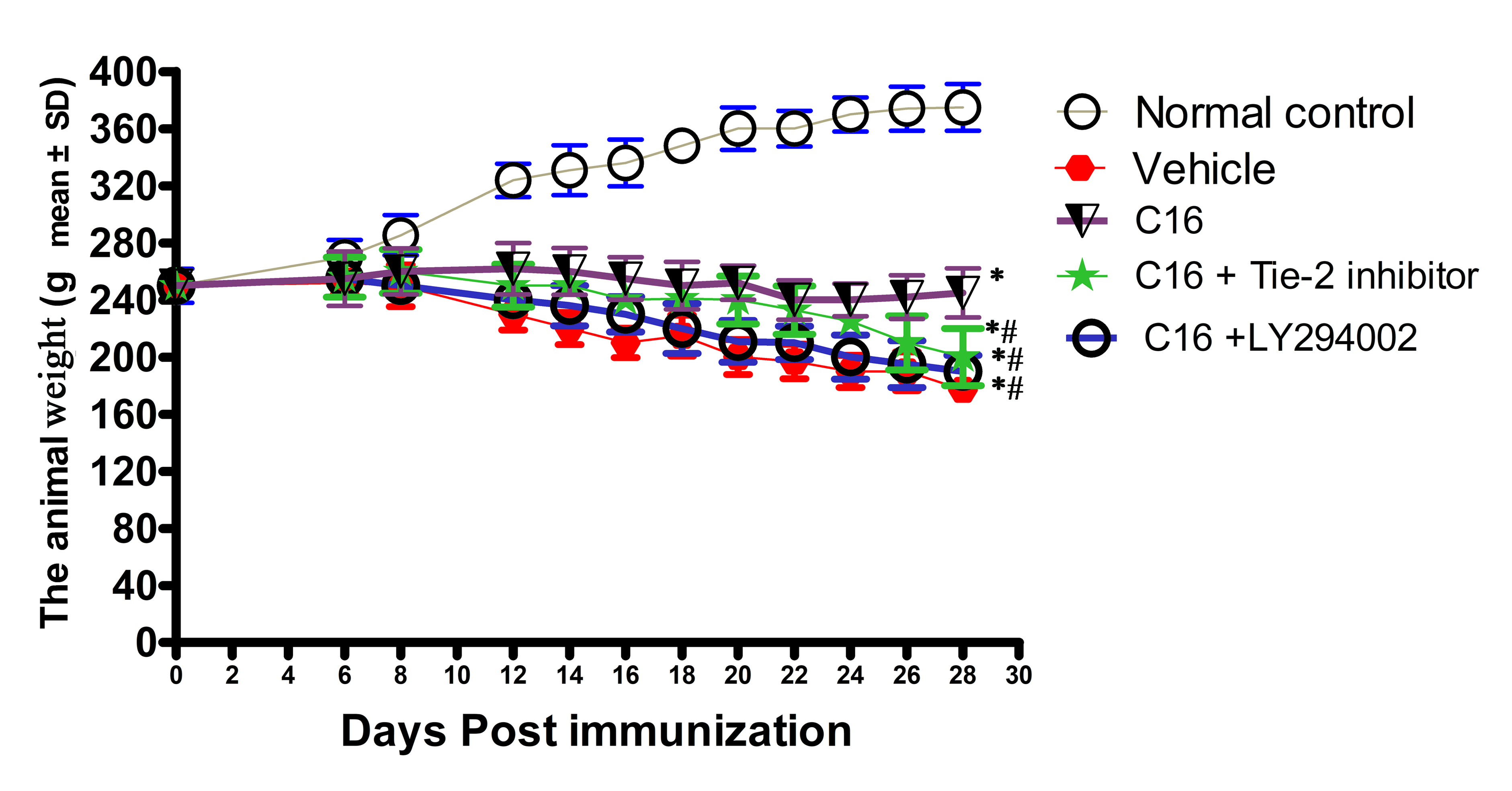

Supplement: Supplementary file 3 [file Image3.TIF]

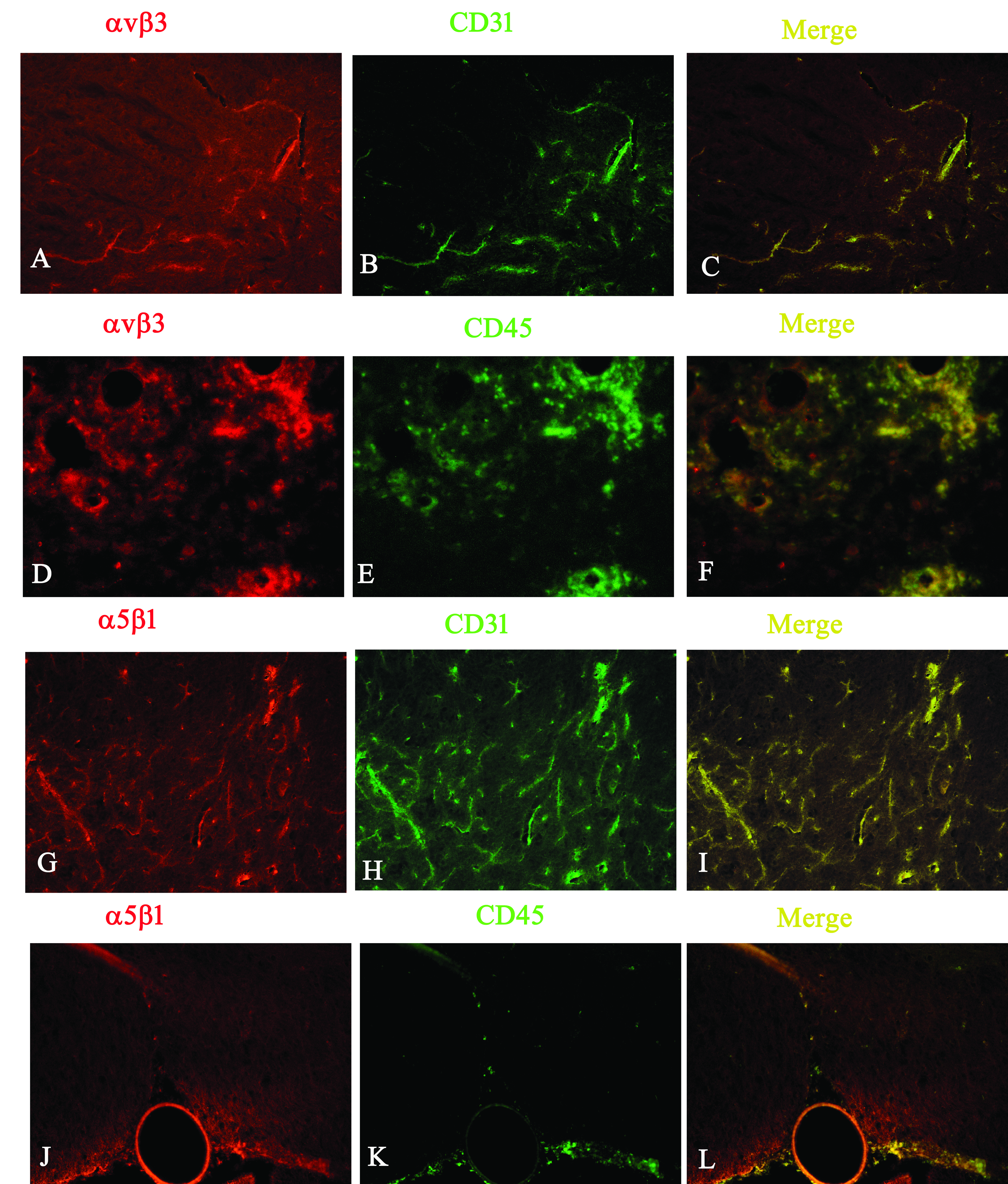

Supplement: Supplementary file 4 [file Image1.JPEG]

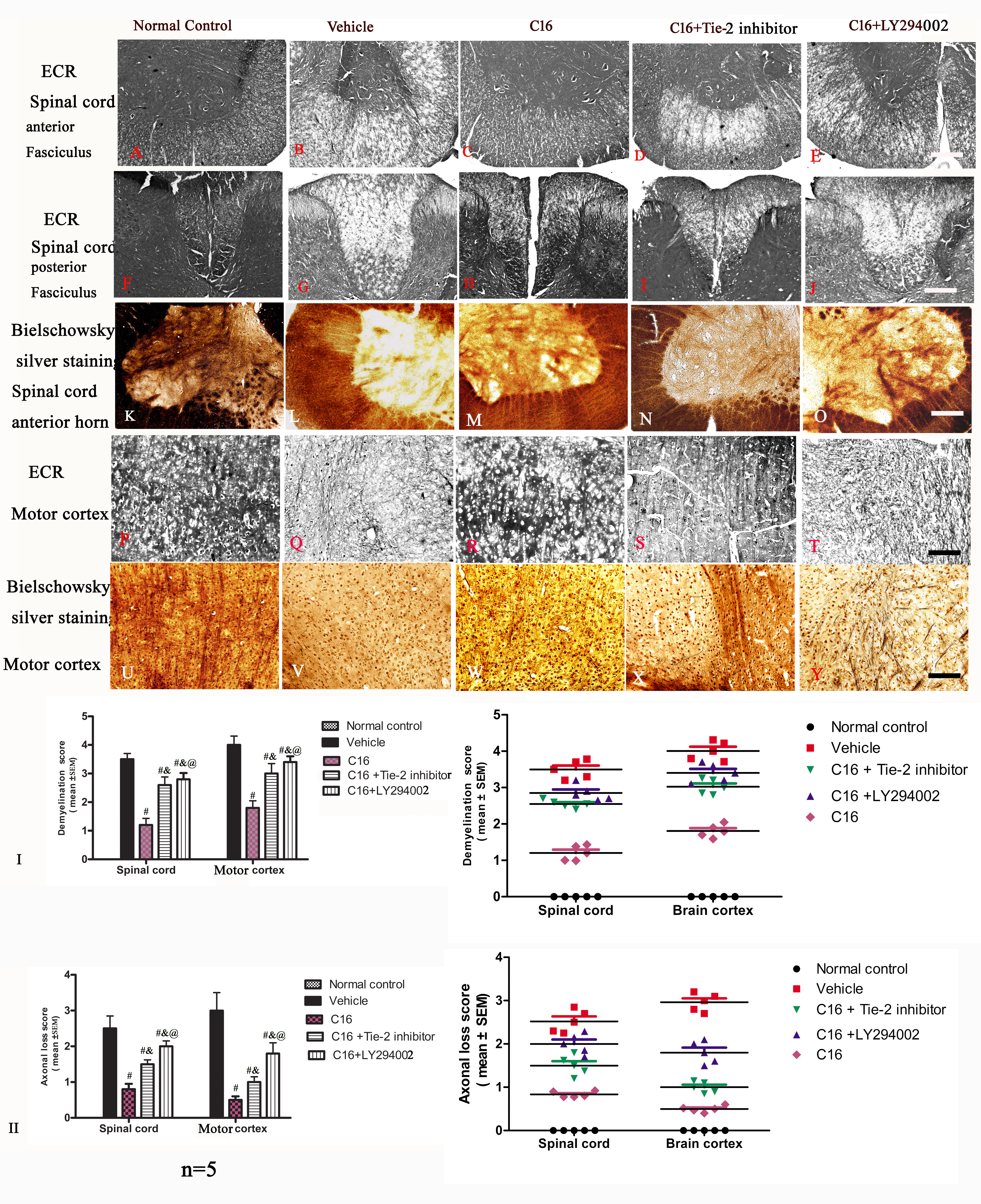

Supplement: Supplementary file 5 [file Image4.JPEG]

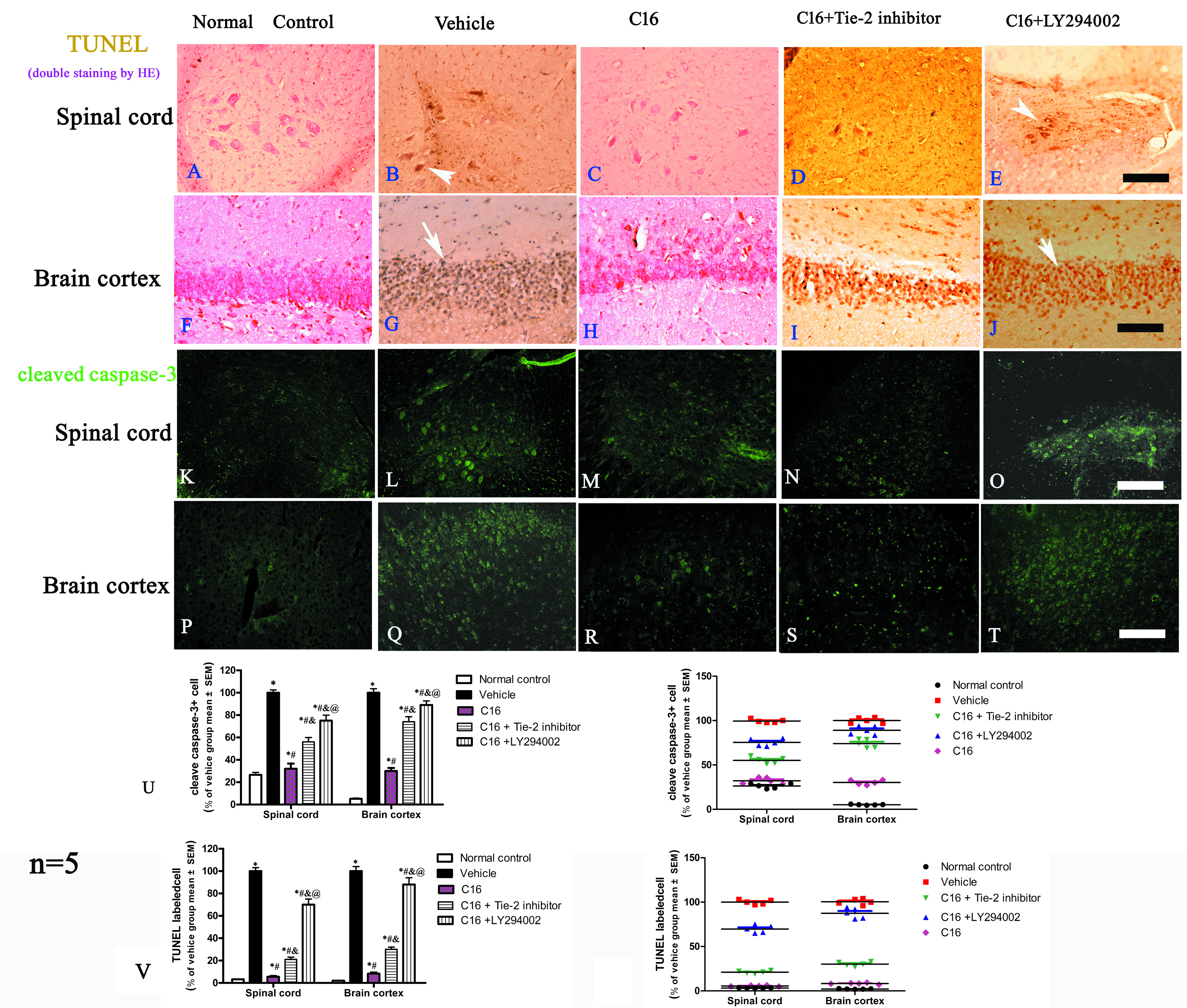

Supplement: Supplementary file 6 [file Image7.JPEG]

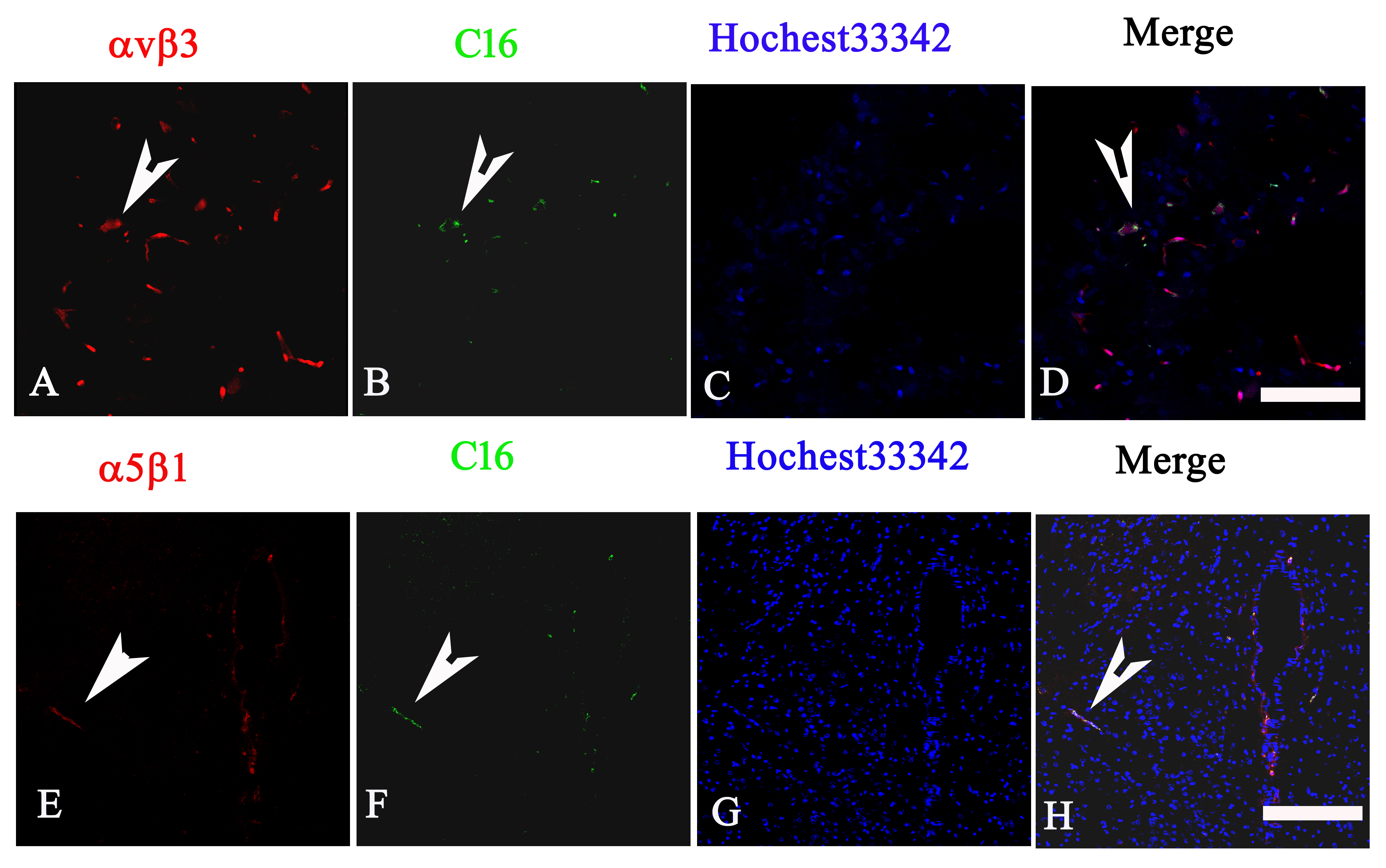

Supplement: Supplementary file 7 [file Image2.JPEG]

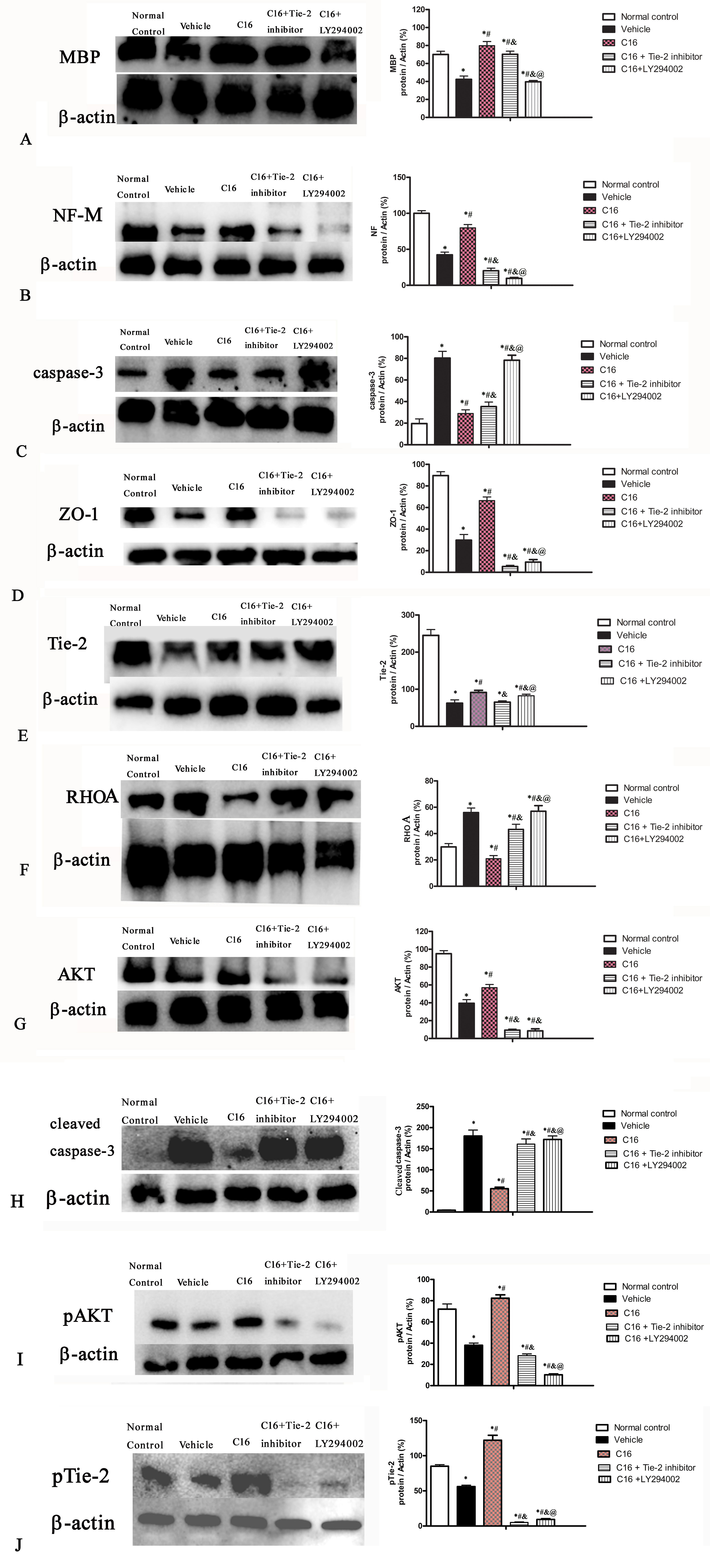

Supplement: Supplementary file 8 [file Image5.jpeg]

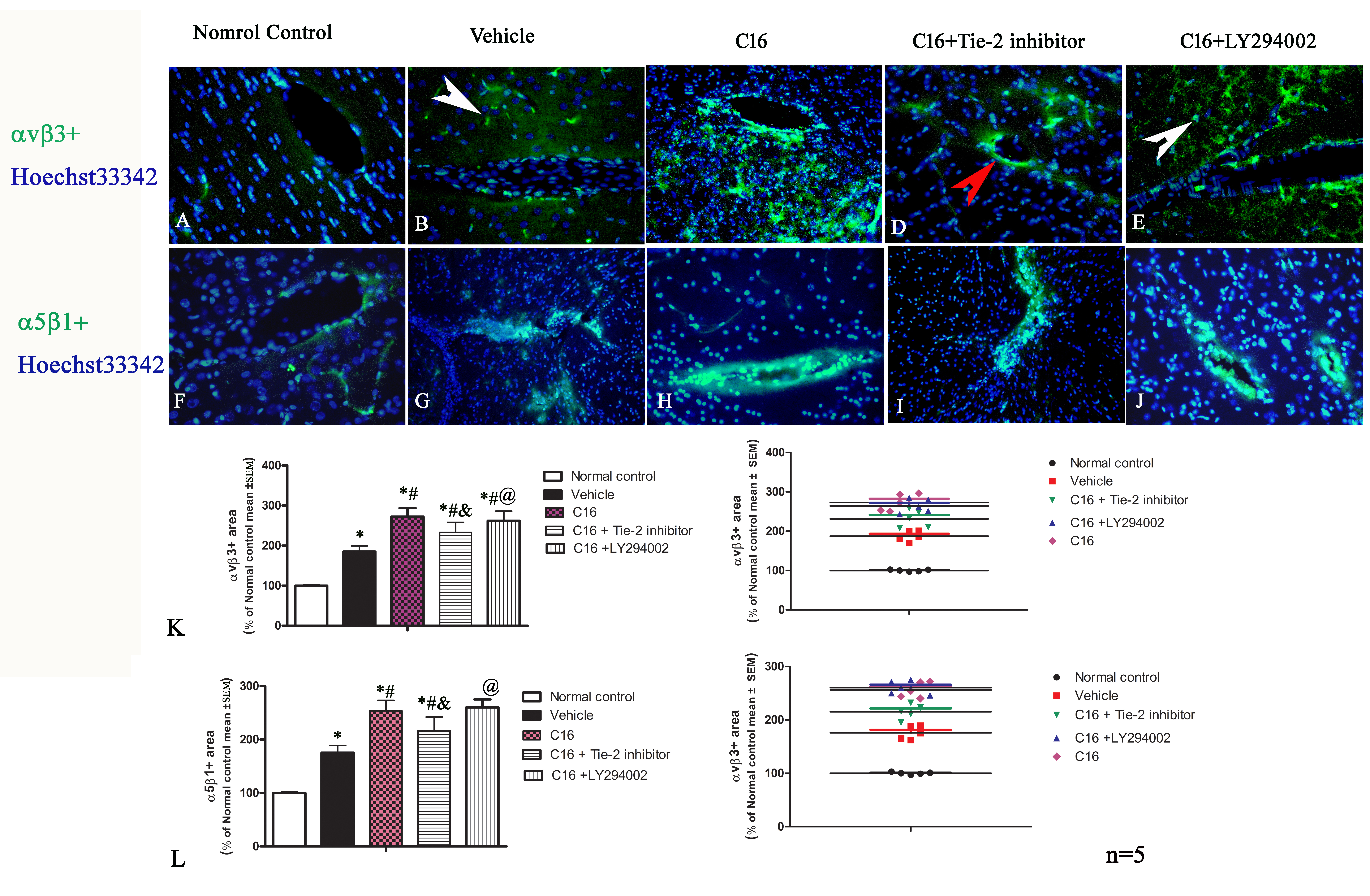

Supplement: Supplementary file 9 [file Image10.JPEG]

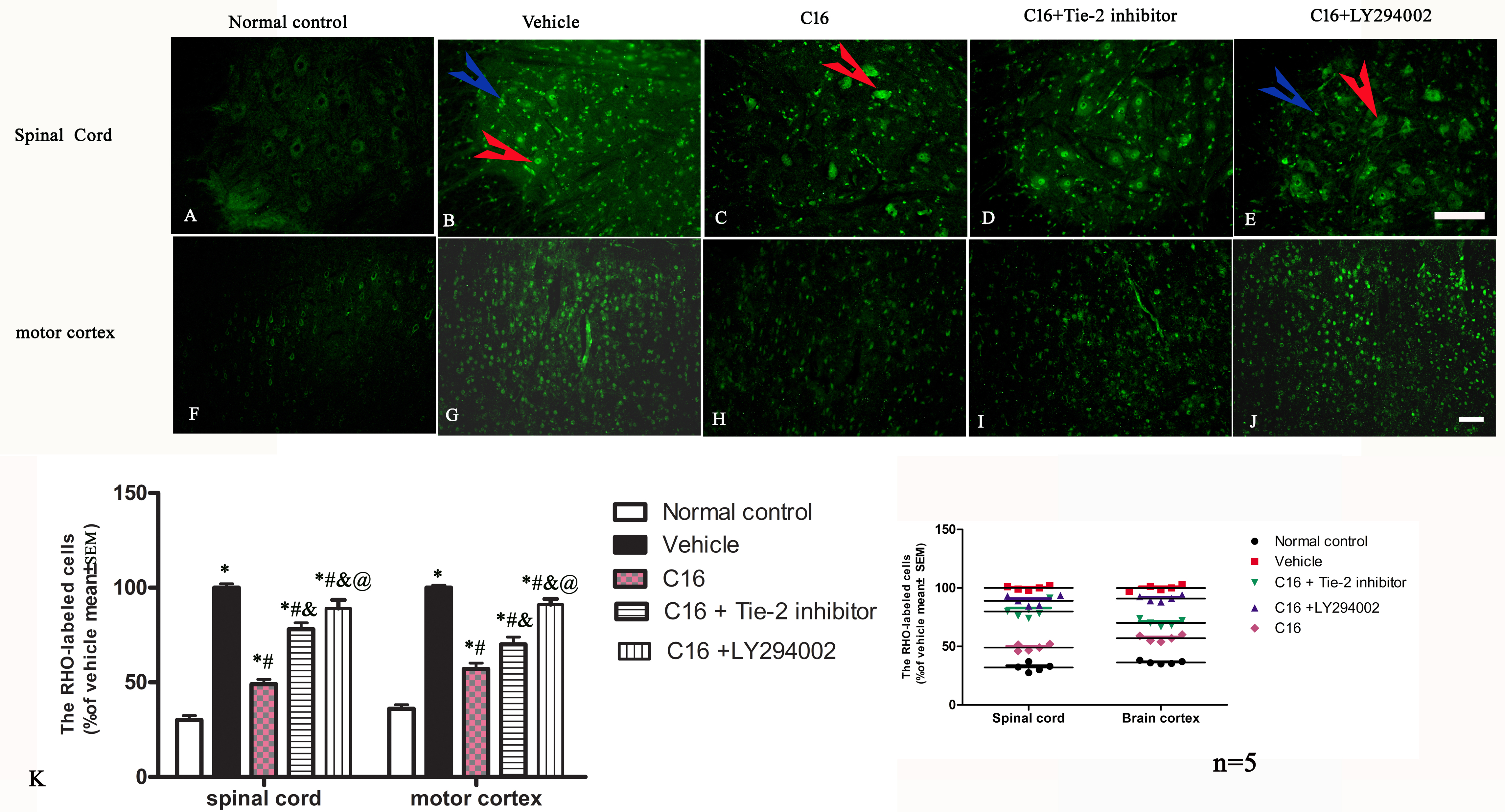

Supplement: Supplementary file 10 [file Image8.JPEG]

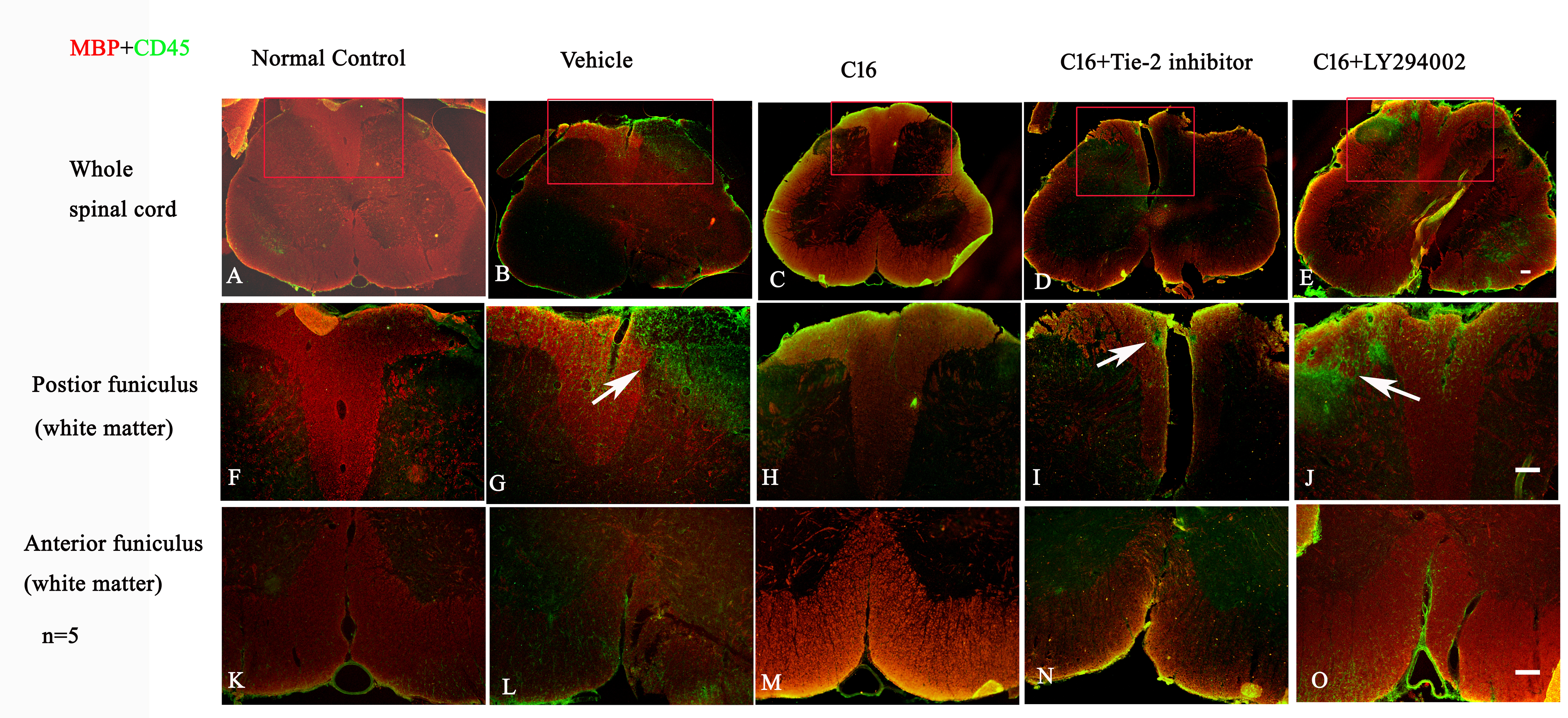

Supplement: Supplementary file 11 [file Image6.JPEG]
